# Profiling with senescence-associated secretory phenotype score identifies GDC-0879 as a small molecule sensitizing glioblastoma to anti-PD1

**DOI:** 10.1038/s41419-025-07915-3

**Published:** 2025-08-09

**Authors:** Yang Liu, Yuan Feng, Lin Cheng, Yangxi Xu, Anhua Wu, Peng Cheng

**Affiliations:** 1 Department of Neurosurgery, The First Hospital of China Medical University, Shenyang, Liaoning China; 2 Department of Neurosurgery, Shengjing Hospital of China Medical University, Shenyang, Liaoning China; 3 Department of Neurosurgery, the People’s Hospital of Liaoning Province, Shenyang, Liaoning China; 4 Institute of Health Sciences, China Medical University, Shenyang, Liaoning China

**Keywords:** CNS cancer, Cancer immunotherapy

## Abstract

Senescence-associated secretory phenotype (SASP) in cancer refers to the bioactive secretome produced by senescence cells in the tumor microenvironment, which could be triggered by therapeutics or local stress conditions. Here, we provided a SASP Score in glioblastoma (GBM) with generating a SASP gene panel to identify the potential small molecular candidate targeting SASP in GBM. The effectiveness of this scoring method was firstly interrogated with our in-house GBM cohort and public datasets, including transcriptomic data from bulk and single cell GBM samples. Then, we validate this score with functional assays and transcriptomic profiling of Doxorubicin-induced senescence GBM cells. Multi-omics profiling with this score demonstrates that SASP facilitates IDH wild type glioma progression by modulating both the behaviors of malignant cells and tumor associated macrophages (TAMs), and discloses GDC-0879 as a feasible SASP inhibitor in GBM therapy. Its administration not only attenuates the tumorigenicity of GBM cells, but also sensitizes GBM to anti-PD1 in preclinical mouse models. Together, we provide a SASP evaluation approach in glioma, and use it to highlight the critical role of SASP in GBM progression and demonstrate GDC-0879 as a small molecule impairing GBM tumorigenicity and improving their response to anti-PD1.

**Figure:**
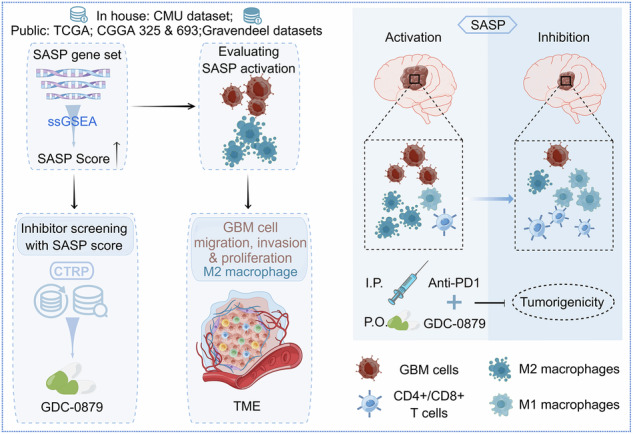
Created by Figdraw (www.figdraw.com).

Created by Figdraw (www.figdraw.com).

## Introduction

As a concomitant phenomenon during aging, cellular senescence is a crucial biological process for tissue regeneration and homeostasis maintenance, which is characterized by sustained cell cycle arrest and could be induced by diverse stress conditions such as low pH, hypoxia, and nutrient deficiency [1]. Senescence-associated secretory phenotype (SASP) is the paracrine signal released from senescence cells and constitutes a characteristic pro-inflammatory secretome. However, emerging evidences demonstrate that this fundamental physiological mechanism could be hijacked by cancer cells for their evolution and immune evasion [2]. When senescence cells present proliferation arrest, the SASP signals released from them provide effective support for cancer progression by modulating the behaviors of residual malignant cells and non-tumor cell components in the tumor microenvironment (TME) to present cancer-promoting phenotypes [3].

To date, glioblastoma (GBM) remains an uncurable malignancy with only 6.8% 5-year overall survival [4]. There is an urgent need for the development of an effective treatment strategies against GBM. Immunotherapy, represented by immune checkpoint blockade (ICB) has revolutionized cancer therapy in the past decade. However, only a few GBM exhibit effective responses to ICB [5], in which the immunosuppressive TME is one of the leading causes limiting the efficiency of ICB in GBM. As a stress response, SASP has paradoxical impacts on the immune surveillance in TME. While it serves as a critical barrier for cancer progression through activating adaptive immunity, the paracrine signals and immune suppressive cytokines released by senescence malignant and non-cancerous cells contribute to the constitution of immune suppressive TME and act as a potential cancer resistance mechanism to immunotherapy [6]. The understanding of multifaceted roles and flexible mechanisms of SASP in GBM may provide potential actionable cancer vulnerabilities for this frustrating disease. Modulating SASP in TME may improve GBM response to immunotherapy. However, the characterization of SASP in GBM progression and its role in regulating the response to immunotherapy remains to be elucidated. Additionally, given the heterogeneity of the cell population exhibiting SASP within the TME, it is essential to dissect the cell components and context-dependent mechanisms concomitant with SASP [7], which remain quite limited.

In the present study, to evaluate SASP activation status in glioma, we generated a SASP gene panel covering the features of senescence processes, including cell cycle arrest, nuclear change, DNA damage response, metabolic adaptation, and increased lysosomal activities, for constructing a SASP evaluation score (SASP Score) by ssGSEA method to stratify glioma patients. Functional assays and validation in multiple cancer senescence datasets demonstrated that the established SASP Score efficiently reflected the status of SASP activation. The application of this evaluation system implicated the involvement of SASP in the dysregulated immune responses and extensive infiltration of tumor-associated macrophages (TAMs) in GBM TME. Screening of small molecular inhibitors based on the SASP Score identified GDC-0879 as a potential antagonist of SASP in glioma, which implication significantly reduced GBM growth and improved their response to PD1 blockade in preclinical mouse models.

## Methods and materials

### Ethics and human specimen collection

This study was approved by the Ethics Committee of Hospital of China Medical University (CMU). The animal experiment protocol was approved and performed under the supervision of the Institutional Animal Care and Use Committee in CMU.

### Dataset availability

In-house CMU cohort incorporates bulk RNA-seq data of 30 cases of grade IV glioma samples, which includes 26 cases of IDH wildtype (wt) and 4 cases of IDH mutant (mut) samples, which is under accession code HRA006353 of the Genome Sequence Archive in National Genomics Data Center, China National Center for Bioinformation/Beijing Institute of Genomics, Chinese Academy of Sciences (https://bigd.big.ac.cn/gsa-human/browse/HRA006353). Glioma RNA-seq data of The Cancer Genome Atlas (TCGA) were obtained from GlioVis data portal (http://recur.bioinfo.cnio.es/), which consists of 428 glioma samples (Grade II 133 cases, grade III 145 cases, and GBM 150 cases) and includes 197 cases of IDH wt and 231 cases of IDH mut glioma samples [8]. Moreover, the RNA-seq data of 921 glioma samples with grade and IDH status information were downloaded from Chinese Glioma Genome Atlas (CGGA) website (http://www.cgga.org.cn), consisting of 312 glioma samples from CGGA325 dataset (Grade II 97 cases, grade III 74 cases, and GBM 137 cases, and 4 cases with not applicable grade information, which includes 145 cases of IDH wt and 167 cases of IDH mut samples) and 609 samples from CGGA693 dataset (Grade II 158 cases, grade III 224 cases, and GBM 227 cases, which includes 276 cases of IDH wt and 333 cases of IDH mut glioma samples). Gravendeel dataset was extracted from UCSC (https://xenabrowser.net/datapages/), including 226 glioma samples with grade information (Grade I 5 cases, grade II 22 cases, grade III 66 cases, GBM 128 cases, and 5 cases of samples with not applicable grade information, which includes 143 cases of IDH wt samples and 83 cases of IDH mut glioma samples). Bulk RNA-seq datasets (GSE271928, GSE266210, GSE274090, GSE121422, GSE158743, GSE208048, GSE212085, GSE130727, GSE240377, and GSE78220) were also obtained from Gene Expression Omnibus (GEO) database (https://www.ncbi.nlm.nih.gov/gds). GSE271928, GSE266210, GSE274090, GSE121422, GSE158743, GSE208048, GSE212085, and GSE130727 were employed for the validation of SASP Score. GSE78220 was applied for the immunotherapy response and survival analysis. Van-Allen dataset was derived from the study “Genomic correlates of response to CTLA-4 blockade in metastatic melanoma” [9]. IMvigor210 CoreBiologies data was downloaded with the R package provided by the following website (http://research-pub.gene.com/IMvigor210CoreBiologies/packageVersions/). RNA-seq data for the SASP Score evaluation of GBM samples receiving TTF treatment was downloaded from the supplement information of Xu’s paper [10].

The analysis of single-cell RNA-seq (scRNA-seq) GBM datasets were derived from GSE235676 containing 149,048 cells from 24 GBM samples (5 newly diagnosed, 7 recurrent, and 12 receiving neoadjuvant combination therapy of pembrolizumab and anlotinib) [11], GSE131928 containing 7930 single cell samples from 28 cases of GBM [12], and GSE84465 containing 3589 single cell data from 4 GBM samples, respectively [13]. All of these three scRNA-seq datasets were downloaded from GEO database (https://www.ncbi.nlm.nih.gov/gds). CGGA scRNA-seq data [14] were extracted from CGGA website (http://www.cgga.org.cn/download.jsp).

### Generation of gene panel for SASP evaluation in Glioma

The detailed information of SASP gene set (91 genes) generated in the present study and related references was listed in Table S1. The SASP gene set was derived by performing an in-depth, rigorous literature search to predict the characteristics of SASPs (Table S1), and predominantly consisted of SASP factors (*n* = 77), while including senescence markers (*n* = 14) covering different features of senescence (cell cycle arrest-associated factors, DNA damage response-associated factors, metabolic adaptation-associated factor, increased lysosomal content-associated factors, and nuclear change-associated factors) and more common in the central nervous system (CNS).

### Establishment of SASP score

The SASP Score for each sample was calculated using the R package “GSVA” with the ssGSEA method, based on a predefined gene panel (Table S1) [15].

### Cell culture

The primary patient-derived glioma stem-like cell (GSC0117) and adherent GBM cell (PGC62) used in this study were isolated from fresh surgical GBM specimens immediately after tumor resection [16]. GSC0117 was cultured in Dulbecco’s modified Eagle’s medium (DMEM)/F12 medium (10565018, Gibco) containing 2% B27 supplement (175004044, Gibco), 20 ng/ml epidermal growth factor (EGF, AF-100-14, Peprotech), 20 ng/ml basic fibroblast growth factor (AF-100-18B, Peprotech), and 2.5 μg/ml heparin (H3149, Sigma). PGC62 and THP-1 (National Collection of Authenticated Cell Cultures, China, TCHu 57) were maintained in RPMI-1640 medium (RPMI) with 10% fetal bovine serum (FBS) (Gibco) and 1% penicillin/ streptomycin (Gibco). GL261 cells were maintained in DMEM (10566024, Gibco) supplemented with 10% FBS and 1% penicillin/ streptomycin.

Mouse bone marrow-derived macrophages (BMDMs) were derived from C57BL/6 mice [16]. Briefly, after the mice were euthanized, the femur and tibia were removed and then bone marrow was flushed out with RPMI-1640 medium. The suspension was passed through a 70 μm stainless steel mesh and erythrocytes were lysed with red blood cell lysis buffer. Then, cells were collected by centrifugation and cultured in RPMI-1640 culture medium supplemented with 10% FBS and pre-charged with 20 ng/ml macrophage colony stimulating factor (416-ML, R&D) for 2–3 days to induce mouse BMDMs. Mouse GBM cells (mGSCs) were isolated from spontaneous GBM tissues established by Sleeping Beauty (SB) transposon technique as previously described [17] and cultured in stem cell medium (Neurobasal-A medium with the addition of B27, 10 ng/ml EGF, and 10 ng/ml FGF) at 37 °C in 5% CO_2_.

### RT-qPCR

After sample collection, total RNA was isolated with TRIzol™ Reagent [16], and then was reversely transcribed into cDNA with PrimeScript™ RT Master Mix (Takara). qPCR was performed with TB Green® Premix Ex Taq™ II (RR820A, TaKaRa). The mRNA expression of target genes was calculated by 2^−ΔΔCt^ method [18] and normalized to 18S mRNA expression. The sequences of RT-qPCR primers were listed in Table S2.

### Bulk RNA-sequencing (Bulk-seq)

The total amount and integrity of RNA were assessed by the RNA Nano 6000 Assay Kit from the Bioanalyzer 2100 System (Agilent Technologies). Library fragments were purified using an AMPure XP system (Beckman Coulter). The insert size of the libraries was detected using an Agilent Bioanalyzer 2100. When the libraries were qualified, the different libraries were sequenced by Illumina NovaSeq 6000. All procedures were performed according to the manufacturer’s instructions. The differentially expressed genes (DEGs) was acquired using the ‘DEseq2’ R package (|log 2 FC| > 1 and *P*.adj < 0.05).

### Small molecule screening

Small molecule screening was performed following the described experimental procedures [19]. Briefly, to recognize specific gene-drug interactions of glioma cells, the data of 30 glioma cell lines were retrieved from the web portal of Cancer Cell Line Encyclopedia (CCLE) project (https://portals.broadinstitute.org/ccle). After measuring the expression levels of SASP genes [20], SASP Scores of each glioma cell line were calculated with the ssGSEA method. Then, the small molecular screening data (drug sensitivity profiles and glioma cell lines) were obtained from the Cancer Therapeutics Response Portal (CTRP project) [21, 22]. Area Under Curve (AUC) was employed to represent drug response, and a large or small AUC value was defined as resistant or sensitive, respectively. Spearman correlation analysis between SASP Score and AUC values of drug response was performed, and drug candidates with significant negative correlation (representing the sensitivity of drugs to SASP) were ranked. Finally, drugs with lower negative correlation values were supposed as potential SASP inhibitors of glioma cells.

### Senescence induction and senescence-associated β-galactosidase staining

Senescence of indicated cell samples was induced by Doxorubicin treatment (200 nM) for 48 h [23]. Then, senescence cell samples were collected at the 5th day after senescence induction and applied for the following experiments as indicated. The senescence induction of cell samples was confirmed by senescence-associated β-galactosidase staining experiments, which was performed with a senescence-associated β-galactosidase staining kit (Beyotime Institute of Biotechnology) following the manufacturer’s protocol.

### Cell proliferation assays

After cell dissociation, single cell suspension of indicated cell samples (5 × 10^3^ cells in 200 μl medium per well) were seeded into 96-well plates. After the indicated treatment, cell growth was measured according to the manufacturer’s instructions at indicated time points by adding 20 μl of MTS solution (G3581, Promega) to the wells and followed by 3 h incubation at 37 °C. The optical densities (OD) of each well were measured with a VICTOR Nivo Multi-Mode microplate reader at an absorbance of 490 nm.

### Co-culture experiments

The co-culturing system was established using six well transwell inserts with 0.4 µm pore size (3450, Corning). The senescence cells were obtained by doxorubicin induction as mentioned above. For co-culture experiments, senescence induced or control cells were seeding into the transwell inserts and received GDC-0879 10 µM or DMSO treatment for 48 h. Then, the inserts containing 2 × 10^5^ control or senescence induced cells were transferred into the wells of 6-well plate, which was pre-seeded with 2 × 10^5^ untreated GBM cells. Then, after 48 h co-culture, GBM cells in the lower chambers were collected for migration, invasion, and proliferation assays.

For co-culture experiments of macrophages and untreated GBM cells, 5 nM PMA was firstly added into transwell inserts containing 2 × 10^5^ THP-1 cells to acquire macrophages.

### In vitro cell migration and invasion assays

Transwell chambers with a pore size of 8 μm (Corning, 3422) were used for cell migration and invasion assay in vitro [16]. GBM cells after co-cultured with indicated GBM cells or macrophages for 48 h were resuspended in culture medium containing 0.2% FBS. Then, 200 µl cell suspension containing 1 × 10^5^ cells (for migration assays) or 2 × 10^5^ cells (for invasion assays) was seeded into the upper chambers of transwell inserts. Then, 600 µl of culture medium containing 20% FBS (for migration assays) or conditioned medium (CM) from indicated cell samples (for invasion assays) was added into the lower chamber. After 24 h co-culture, the chamber membranes were fixed with methanol and stained with 0.1% crystal violet solution. Cells on the upper side of the membrane were removed with a cotton swab, and cells migrating or invading to the lower side of the membrane were observed and photographed with an upright microscope.

### Immunohistochemical (IHC) staining

IHC staining was performed according to the following procedures. After antigen retrieval and endogenous peroxidase blocking, the slides were treated with 3% bovine serum albumin, and then incubated with the corresponding primary antibody at 4 °C overnight, followed by secondary antibody treatment and DAB visualization. Protein expressions were quantified by German Immunohistochemical Score [24].

### Flow cytometry (FACS)

The percentage of apoptotic cells in cell samples treated with GDC-0879 or Doxorubicin were detected by FACS with FITC Annexin V Apoptosis Detection Kit (BD Pharmingen), according to the manufacturer’s guidance. FACS analysis of orthotopic GBM tissues was performed following the protocol described previously [25, 26]. FlowJo software was applied for the analysis of FACS data.

### Immunoblotting

Cell samples were collected and lysed with RIPA buffer containing PMSF (10:1) to extract protein. After protein quantification, each sample with equal micrograms of protein was loaded into a lane and followed by electrophoresis. Subsequently, the protein was electrotransferred to a 0.45 μm polyvinylidene difluoride membrane, which was blocked with 5% skimmed milk and incubated with the primary antibody solution at 4 °C overnight. Then, secondary antibodies were added to incubate the membranes at room temperature for 1 h. Protein bands were visualized with a chemiluminescence ECL reagent. All immunoblot results were independently repeated for at least three times and the antibodies are listed in Table S4.

### Immunofluorescence staining

Tyrosine Signal Amplification (TSA) technology was employed for simultaneous labelling of multiple proteins on the same sample section (human GBM or mouse GBM samples), which enables quantitative immunofluorescence analysis of indicated proteins. Briefly, after gradient hydration of the sections, antigen repair and H_2_O_2_ blocking were performed. The sections were further incubated with 3% BSA, and after gently shaking off the blocking solution, the primary antibody solution was added to the sections (The information of the antibodies are shown in Table S4). After overnight incubation, the corresponding kind of HRP-labelled secondary antibody was added and washed three times with PBS. Then, the corresponding TSA was added and incubated for 10 min. After being washed with PBS three times, the sections were heated in a microwave oven to remove the primary and secondary antibodies that had been bound to the tissue. Then, the addition of the corresponding primary antibody-secondary antibody-TSA-microwave heating was repeated to complete the indicated TSA staining. Finally, the slides were washed, re-stained with DAPI, and blocked to obtain images. Immunofluorescence images were acquired by Pannoramic MIDI (3DHISTECH) and CaseViewer.

For the analysis of immunofluorescence images with TSA labelling [27], the percentages of senescence malignant cells (P21^+^/γ-H2AX^+^/Lamin B1^−^/GFAP^+^), senescence macrophages (P21^+^/γ-H2AX^+^/Lamin B1^−^/IBA1^+^), or senescence M2 macrophages (P21^+^/γ-H2AX^+^/Lamin B1^−^/CD163^+^) cells were separately calculated by counting the number of GFAP^+^, IBA1^+^ or CD163^+^ cells that also co-stained with P21^+^/γ-H2AX^+^/Lamin B1^−^ per visual field (human GBM samples, ×80). The mouse GBM samples are also analyzed using the same methods as human GBM samples (per visual field, ×125). For apoptotic cells, the percentages of apoptotic malignant cells (Cleaved casepase3^+^/GFAP^+^), and apoptotic macrophages (Cleaved casepase3^+^/ IBA1^+^) were individually estimated by counting the number of GFAP^+^ and IBA1^+^ cells that also co-stained with Cleaved casepase3^+^ per visual field (×125). The total number of cells in three non-overlapping high-power fields was counted from randomly selected human or mouse GBM samples. Three replicates were performed for each group.

### Mouse intracranial xenograft model

To establish mouse orthotopic GBM xenograft model, C57BL/6 mouse was stereotactically injected with 5 × 10^5^ GL261 cells or 5 × 10^4^ mGSCs obtained from SB transposon-derived mouse GBM sample with primary culture technique into the right cerebral cortex at a depth of 3.5 mm [16]. Then, the mice were randomly assigned into 4 groups: Control: received a single oral dose of 0.5% methylcellulose/0.2% Tween 80 (MCT) and IgG2b isotype control; GDC-0879: GDC-0879 (100 μg/g body weight) in MCT was orally administered by gavage daily from 7th to 21st day; Anti-PD1: Anti-PD1 antibody (10 μg/g body weight) was injected intraperitoneally on the 5th, 8th, and 11th day; Combined treatment: treated with GDC-0879 and Anti-PD1). Intracranial tumor formation was confirmed by bioluminescence assay on the 7th day after the implantation of GL-261 and mouse GBM cells. Anti-PD1 antibody (10 μg/g body weight) was injected intraperitoneally on the 5th, 8th, and 11th day [16]. GDC-0879 (100 μg/g body weight) was orally administered by gavage daily from 7th to 21st day. The mice were executed on day 21 for FACS and IHC analysis, or observed to present neurological symptoms and then sacrificed for survival analysis.

## Bioinformatic analysis

### Gene Ontology (GO), Kyoto Encyclopedia of Genes and Genomes (KEGG), and Gene Set Enrichment Analysis (GSEA)

GO biological processes (BP) and KEGG analysis were performed based on the genes positively related to SASP Score (*R* ≥ 0.6, Spearman correlation analysis) with IDH wt glioma samples from indicated datasets by DAVID (https://david.ncifcrf.gov/) [28]. GSEA (http://www.broadinstitute.org/gsea/index.jsp) was conducted to analyze the enrichment of indicated phenotypes between high and low SASP Score groups using the median SASP Score as a threshold. Normalized enrichment score (NES) and false discovery rate were used to determine the statistical significance.

### Tracking tumor immunophenotype (TIP) analysis

TIP analysis was performed with the website tool (http://biocc.hrbmu.edu.cn/TIP/index.jsp), according to the protocol provided [29]. TCGA IDH wt glioma transcriptome expression profile was used as an input file for the online analysis. The score of different immune cells recruited in Step 4 were summed to derive a total score for “Step 4: Trafficking of immune cells to tumor”.

### Immune cell component estimation with Xcell and Cibersort

R package “Estimate” was used to evaluate tumor purity in the TME and calculate the Immune, Stromal, and Estimate scores in TCGA IDH wt glioma dataset [30]. The abundance of immune cells in IDH wt glioma samples of TCGA/CGGA325/CGGA693/Gravendeel datasets was quantified by Xcell (https://xcell.ucsf.edu/) [31], Microenvironment cell populations (MCP)-counter [32], and ssGSEA [15, 33].

### Statistical analysis

All statistical analyses were performed using Microsoft Excel 2019 or Prism 8 unless indicated otherwise. The data were expressed as Mean ± S.D., and two-tailed *t*-test or one-way ANOVA (Turkey’s post-test) was employed to determine the differences between two or among more groups, respectively. *P* < 0.05 was considered to be statistically significant. Univariate and Multivariate Cox regression analyses were performed by R package “Survival”. Hazard Ratio (HR) calculated > 1 was considered as risky factor, while HR < 1 was regarded as protective factor. Kaplan–Meier survival curves and the log-rank test were employed to evaluate the prognostic significance. R package “pheatmap” was used for making heat maps by R (version 4.2.1). R package “GSVA” (http://www.bioconductor.org) was used to assess the underlying pathway activity variation according to the gene-sets of defined signaling pathways. R package “Maftools” was employed to illustrate significant mutation information of SASP-related genes.

## Result

### Establishing SASP Score to evaluate SASP activation in glioma

**Fig. 1:**
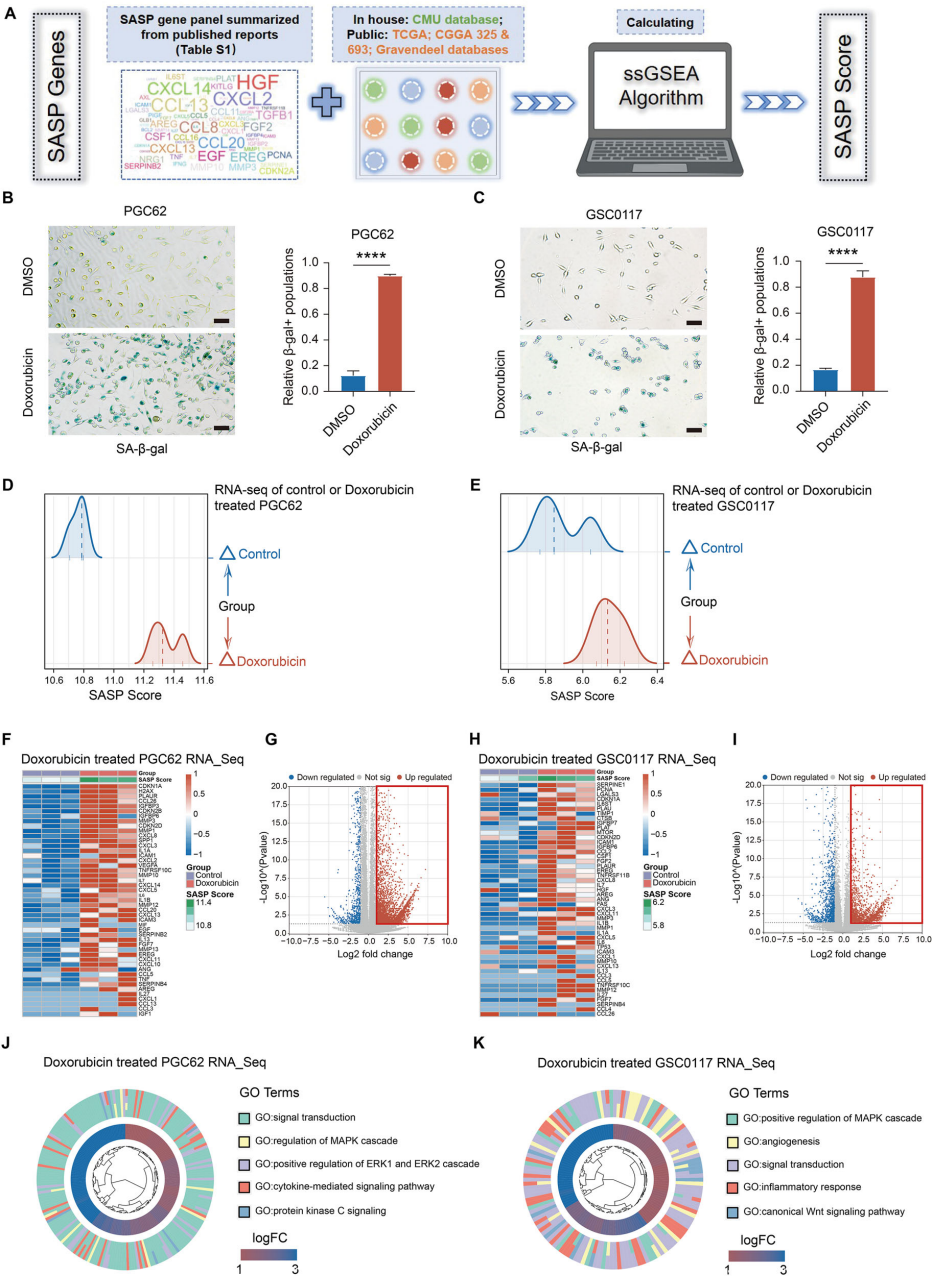
Establishment of SASP Score reflects SASP activation in cancer. **A** The schematic graph showing the establishing procedures of SASP Score with ssGSEA method and the SASP gene panel summarized from published reports according to senescence hallmarks listed in Table S1. **B**, **C** Representative SA-β-gal staining images of indicated GBM cells (**B**: PGC62 and **C**: GSC0117) with control (DMSO) or Doxorubicin (200 nM) (*n* = 3, *t*-test). Scale bar, 50 μm. **D**, **E** The analysis of RNA-seq data obtained from PGC62 (**D**) and GSC0117 (**E**) samples with control (DMSO) or Doxorubicin (200 nM) treatment indicating the elevation of SASP Score accompanied with senescence induction by Doxorubicin treatment in these cells (*n* = 3). **F**, **H** The analysis of RNA-seq data obtained from PGC62 (**F**) and GSC0117 (**H**) samples with control (DMSO) or Doxorubicin (200 nM) treatment showing the elevation of SASP Score reflects the upregulation of SASP associated genes. **G**, **I** The analysis of differentially expressed genes (DEGs) according to RNA-seq data obtained from PGC62 (**G**) and GSC0117 (**I**) samples with control (DMSO) or Doxorubicin (200 nM) treatment (with DESeq2). **J**, **K** Gene Ontology (GO) biological process analysis with RNA-seq data obtained from PGC62 (**J**) and GSC0117 (**K**) samples with control (DMSO) or Doxorubicin (200 nM) treatment. (**** *P* < 0.0001).

Considering the potential versatile roles of cellular senescence and SASP in cancer biology, we firstly sought to summarize an optimized SASP panel for the comparative evaluation of SASP status in glioma. Given the context-dependent nature of SASP and the defects of single senescence marker, we generated a characterized SASP gene set containing 91 genes through literature inquiry, including recent SenNet paper (Table S1). Our SASP gene panel incorporated 77 genes encoding classical SASP factors (Table S1; including 20 chemokines, 21 growth factors and regulators, 18 proteases and regulators, 9 soluble or shed receptors or ligands, 7 interleukins, and 2 other inflammatory factors). Moreover, it has been previously reported that, in the absence of cell-cycle arrest (non-senescence state), brain cells also secrete inflammatory molecules that overlap with SASP factors [34]. Thus, to circumvent the possibility of misidentifying the SASP gene set as inflammatory components normally detected in cells rather than a critical SASP, we also brought 14 well-recognized senescence markers into our gene set. These markers not only cover the classical senescence markers of CNS, but also include the molecules reflecting the hallmarks of cancer senescence including cell cycle arrest (CDKN1A, CDKN2A, CDKN2B, CDKN2D, MTOR, PCNA, SATB1, and TP53), DNA damage response (MACROH2A1), anti-apoptotic pathway activation (BCL2), increased lysosomal content (GLB1, LGALS3, and LGALS3BP), and nuclear change (LMNB1) (Table S1). Then, we employed ssGSEA method to develop an SASP Score to evaluate the status of SASP activation in glioma samples (Fig. 1A and Table S3) [15].

Next, considering the heterogeneity of senescence in different tissues, we collected 8 senescence GEO datasets and compared our SASP Score with 4 other senescence signatures, including SenMayo [35–38]. These 8 senescence-related GEO datasets encompass 9 distinct induction strategies for cellular or tissue senescence, including both tumor and non-tumor models: GSE271928, radiation-induced senescence in GBM cell lines A172 and LN229; GSE266210, p16_high (senescence) and p16_low (non-senescence) in GBM tissues; GSE274090, radiation-induced senescence in U251 GBM cells; GSE121422, radiation-induced senescence in LN229 GBM cells; GSE158743, radiation-induced senescence in U2OS human osteosarcoma cells; GSE208048, palbociclib-induced senescence in SK-MEL-103 melanoma cells; GSE212085, doxorubicin-induced senescence in IMR90 fibroblast cells; GSE130727, doxorubicin-induced senescence in WI-38 fibroblast cells. We established the individual senescence scores for our SASP panel and the other four signatures by ssGSEA in the senescence datasets, respectively, and compared them with the non-senescence control models. Our analysis revealed that five established senescence signatures exhibited marked upregulation in the majority of senescence models. However, with the exception of our SASP Score, none of the existing signatures (including SenMayo) demonstrated consistent upregulation across all nine senescence induction paradigms (Fig. S1A). This may be attributable to the heterogeneity of senescence phenotypes and SASP composition across tissues. These findings highlight the superiority of our SASP Score in achieving better efficacy in senescence characterization among different senescence models. Collectively, our data indicate that SASP scoring with the current SASP gene panel and ssGSEA method serves as a suitable approach to evaluate the status of SASP activation in glioma.

### Functional experiments in GBM cells reveal that the SASP Score efficiently represents the status of SASP activation

Next, we also sought to test whether the elevation of this SASP score effectively represented SASP activation. We firstly validated our SASP scoring method in a dataset (GSE240377) which includes the data obtained from four senescence-induced GBM cell lines by TMZ or radiotherapy [39]. The result demonstrated that SASP Score was significantly up-regulated in TMZ/radiotherapy-induced senescence GBM cells (Fig. S2A). This supports the representative of our SASP score in the evaluation of glioma senescence. Then, we acquired senescence GBM cells with the well-recognized Doxorubicin-induced senescence method [23]. Senescence in two primary GBM cell lines (primary adherent GBM cell PGC62 and GBM stem-like cell GSC0117, Table S4) was induced by 200 nM Doxorubicin treatment and confirmed by the expression validation of two well-recognized senescence markers, SA-β-gal and P21. We observed enhanced intensities of SA-β-gal staining and elevated mRNA expression of P21 after Doxorubicin treatment in these cells (Figs. 1B, C and S2B, C). Moreover, qPCR analysis disclosed that mRNA expression of SASP factors in these cells (IL6, IL1α, IL1β, TNFα, CXCL10, MMP3, CXCL10, and CCL2) were increased with Doxorubicin treatment (Fig. S2B, C). These results support the successful senescence induction in these two GBM cell lines. Thus, we employed these cell samples to evaluate the representative of our SASP scoring method for SASP activation. Indeed, ssGSEA analysis of RNA-seq data obtained from these samples showed a significant elevation of the SASP Score in Doxorubicin-treated cells compared to control samples (Fig. 1D, E). Subsequent heatmap analysis revealed that the upregulation of our SASP Score efficiently reflected the elevation of SASP-related gene expression in these two GBM cell lines (PGC62, Fig. 1F; GSC0117, Fig. 1H). The enrichment analysis of DEGs between Doxorubicin-treated and control samples demonstrated that the DEGs were mainly enriched in signal transduction and oncogenic pathways like MAPK cascade (DEGs: Fig. 1G, I and Table S5; GO: Fig. 1J, K; KEGG: Fig. S2D, E).

Given that SASP includes a range of pro-inflammatory factors [40, 41], we sought to distinguish whether the elevation of our SASP Score reflects the activation of SASP rather than inflammatory state in cells. We established 4 inflammatory state scores from 4 inflammatory state-associated gene sets obtained from the Molecular Signatures Database [42] (Table S6) by the ssGSEA method. According to analyzing RNA-seq data of Doxorubicin-induced senescence and control PGC62 and GSC0117 samples, the SASP Score was significantly increased in senescent induced samples, whereas the inflammatory state scores didn’t show a consistent elevation in senescence induced samples (Fig. S2F, G). Collectively, these data demonstrated the effectiveness of current SASP panel in representing SASP activation in GBM cells.

### SASP activation is a feature for the malignant progression of IDH wt glioma

**Fig. 2:**
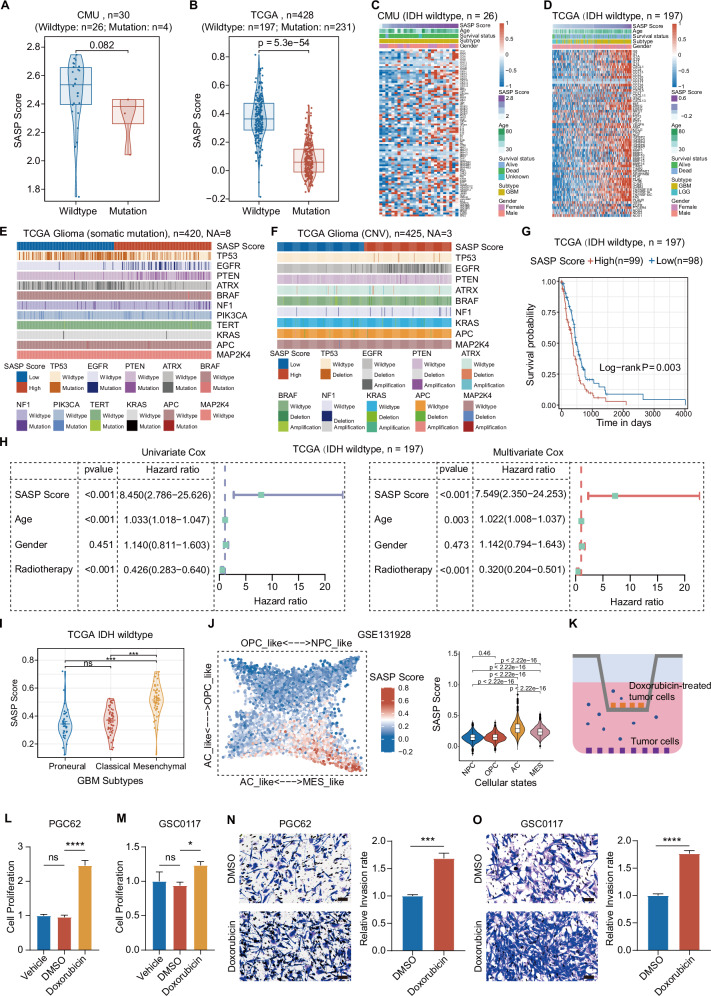
SASP activation is a critical characteristic for the malignant progression of IDH wt glioma. **A**, **B** The SASP Score analysis of IDH wt and mut glioma according to indicated datasets (**A**: CMU GBM, *n* = 30, Wildtype: *n* = 26; Mutation: *n* = 4; **B**: TCGA glioma, *n* = 428, Wildtype: *n* = 197; Mutation: *n* = 231; The samples with unknown IDH status weren’t included in the analysis). **C**, **D** The analysis of CMU and TCGA datasets showing that the elevation of SASP Score reflects the upregulation of SASP associated genes in IDH wt glioma (**C**: CMU IDH wt GBM, *n* = 26; **D**: TCGA IDH wt glioma, *n* = 197). **E**, **F** The characterization of SASP in glioma with established SASP Score based on the indicated key genomic and clinical features of glioma (**E** Somatic mutation: TCGA Glioma, *n* = 420, Not Applicable = 8; **F** Copy number variations (CNV): TCGA Glioma, *n* = 425, Not Applicable = 3). **G** Kaplan–Meier analyses revealing that high SASP Score indicates unfavorable prognosis in IDH wt glioma (TCGA IDH wt glioma *n* = 197: high *n* = 99, low *n* = 98). **H** The univariate (Left) and multivariate analyses (Right) indicating the elevation of SASP Score as an independent prognosis factor of IDH wt glioma (TCGA IDH wt glioma, *n* = 197). **I** The evaluation of SASP Score in indicated subtypes of GBM (TCGA GBM RNA-seq dataset, *n* = 141: proneural *n* = 36, classical *n* = 56, and mesenchymal *n* = 49; Wilcoxon rank sum test). **J** The SASP Score analysis in four GBM malignant cellular states, indicating the enrichment of SASP Score in AC-like and MES-like cellular states, which are significantly higher that other two malignant cellular states, OPC-like and NC-like (GSE131928, *n* = 6576; Left: Two-dimensional representation of cellular states. Each quadrant corresponds to one cellular state, the exact positions of malignant cells (dots) reflects their relative scores for the meta-modules; Right: The analysis of cell-state plots based on SASP Scores of single cell samples in each cell-state; NPC, *n* = 1986; OPC, *n* = 1047; AC, *n* = 1614; MES, *n* = 1929; Wilcoxon test). **K** The schematic graph showing the co-culture method of GBM cells with Doxorubicin (200 nM) treatment (Upper chamber) and untreated GBM cells (Lower chamber). **L, M** MTS assays of indicated GBM cell samples (**L** PGC62; **M** GSC0117; *n* = 3, one-way ANOVA). **N, O** Representative images (Left) and analysis (Right) of Transwell invasion assays of indicated GBM cell samples (**N** PGC62; **O** GSC0117; *n* *=* 3, *t*-test). Scale bar, 50 μm. (ns not significant, ** *P* < 0.01, *** *P* < 0.001, **** *P* < 0.0001).

Due to the paradoxical roles of SASP in cancer, we sought to delineate SASP activation status in glioma and examine the inter-relationship between SASP activation and malignant progression of glioma with our SASP Score. Since IDH status is the most prominent molecular characteristic in glioma, we firstly employed the SASP Score to analyze SASP activation in glioma samples with different IDH status from five glioma bulk-tumor transcriptomic cohorts, including our in-house GBM RNA-seq dataset (Table S7). IDH wt glioma consistently exhibited a significantly higher SASP Score in comparison with IDH mut glioma across all five glioma cohorts, indicating a more active SASP in IDH wt glioma compared to IDH mut glioma. Therefore, we chose IDH wt glioma for further investigation (Figs. 2A, B and S3A). Besides, consistently with the analysis shown in Fig. 1F and H, increased SASP Score efficiently reflected the mRNA upregulation of SASP genes (Figs. 2C, D, and S3B), which indicated that the SASP Score could effectively represent the SASP-related gene expression in IDH wt glioma and could be employed for the evaluation of SASP activation. Through dividing the samples into high and low SASP Score groups using the median SASP Score as a threshold, we observed more EGFR (26.67%), PTEN (27.14%), and NF1 (10%) mutations in the high SASP Score group than low SASP Score group (EGFR (1.90%), PTEN (0.95%), NF1 (4.76%)) of glioma (Fig. 2E). We also observed a few BRAF mutations (1.43%) in the high SASP Score group of glioma (Fig. 2E). In contrast, ATRX mutation (43.81%), which was considered as a molecular signature of low-grade glioma (LGG), was more likely to occur in the low SASP Score group than high SASP Score group (14.76%) (Fig. 2E). Additionally, EGFR amplification (44.60%) and PTEN (8.92%) deletion were more common in the high SASP Score group than low SASP Score group (EGFR 1.89%, PTEN 4.25%) (Fig. 2F) [43]. Interestingly, there were more samples with TP53 mutation in low SASP Score group (54.76%) than high SASP Score group (34.29%) (Fig. 2E), indicating a potential context-dependent role of p53 in regulating glioma senescence.

Next, we examined whether elevated SASP Score indicates poor survival in IDH wt glioma. After the patient samples in the above four IDH wt glioma datasets (TCGA/CGGA325/CGGA693/Gravendeel datasets) were stratified into high and low SASP Score groups with the median value of SASP Score, survival analysis disclosed a consistent shorter patient survival of high SASP Score groups than low SASP Score group in IDH wt glioma (Figs. 2G and S3C). Then, to further distinguish tumor samples based on SASP activation status, we classified all tumor samples using ConsensusClusterPlus method [44], which specified groups with common biological characteristics (Fig. S3D, E). With the selection of *K* = 2 to classify IDH wt glioma samples (TCGA RNA-seq dataset, *n* = 197) into Cluster 1 and 2 (Fig. S3F), principal component analysis (Fig. S3G) and survival evaluation on these two clusters (Fig. S3H) disclosed that Cluster 1 had a significantly better survival trend compared to Cluster 2, while exhibiting a lower SASP Score (Fig. S3I). Additionally, both univariate and multivariate analyses revealed that the elevation of the SASP Score could serve as an independent poor survival predictor for IDH wt glioma, regardless of common clinical characteristics including gender, radiotherapy, and age (Figs. 2H and S4A). Moreover, the analysis between SASP Score and GBM subtypes in TCGA, CGGA325, CGGA693, and Gravendeel four cohorts revealed that the mesenchymal (MES) GBM, the most aggressive GBM subtype [45], exhibited higher SASP Score compared to the Proneural (PN) and Classical (CL) subtypes (Figs. 2I and S4B). Moreover, we evaluated SASP activation status of GBM cells based on four malignant cellular states, which also disclosed an elevated SASP Score in MES-like and AC-like cellular states (Fig. 2J). The analysis of genes positively associated with SASP Score elevation (Table S8, *R* > 0.6, Spearman analysis) through GO BP and KEGG analysis revealed significant enrichment of BP and pathways associated with tumorigenesis and tumor development in high SASP Score groups (Fig. S5A). Lastly, to verify whether SASP strengthened the malignant behaviors of GBM cells like proliferation and invasion, we co-cultured Doxorubicin-induced senescence GBM cells with untreated GBM cells (Fig. 2K). Indeed, senescence GBM cells enhanced the proliferation (Fig. 2L, M) and invasion abilities of untreated GBM cells (Fig. 2N, O), indicating intercellular bioactive mediators released from senescence cancer cells to strengthen the malignant behaviors of GBM cells without senescence induction. Collectively, these data obtained from the analysis based on present SASP Score support SASP activation as a prominent feature accompanying with the malignant progression of IDH wt glioma.

### Elevation of SASP Score indicates dysregulated immune responses and extensive infiltration of macrophages in IDH wt glioma

**Fig. 3:**
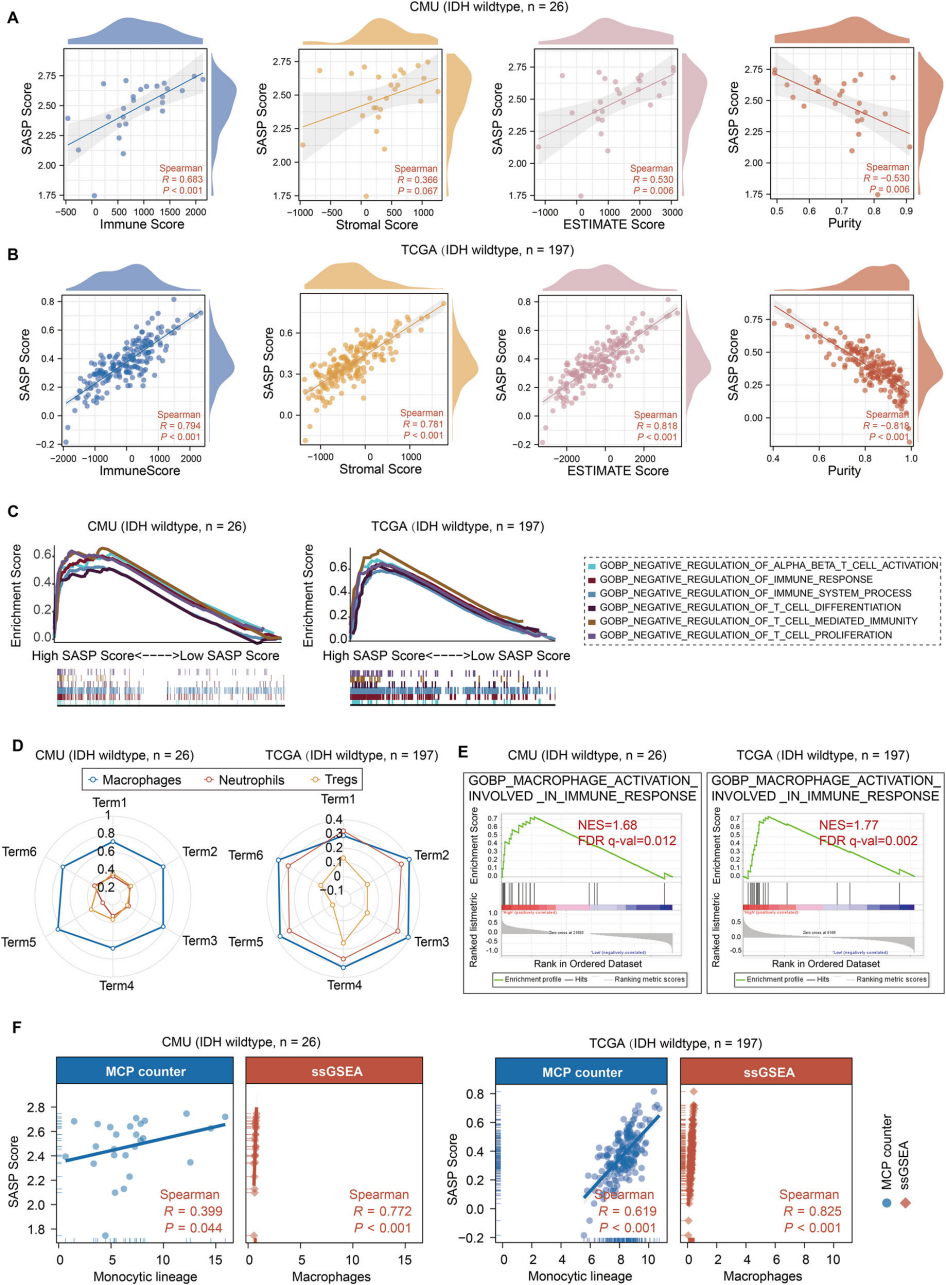
The elevation of SASP Score indicates deregulated immune responses and extensive infiltration of macrophages in GBM. **A**, **B** The correlation analysis of SASP and indicated scores (Left, Immune Score; Middle left, Stromal Score; Middle right, ESTIMATE Score; Right, Purity Score) in CMU IDH wt GBM (**A**, *n* = 26) and TCGA IDH wt glioma samples (**B**, *n* = 197, Spearman correlation analysis). **C** GSEA analysis revealing a potential stronger negative regulation of immune responses in IDH wt glioma samples with high SASP Score than samples with low SASP Score (CMU IDH wt GBM *n* = 26: high *n* = 13; low *n* = 13; TCGA IDH wt glioma *n* = 197: high *n* = 99; low *n* = 98). **D** The correlation analysis of indicated non-malignant immunosuppressive cell populations and indicated immunosuppressive terms in IDH wt glioma (CMU IDH wt GBM *n* = 26; TCGA IDH wt glioma *n* = 197; Term 1: GOBP negative regulation of alpha beta T cell activation; Term 2: GOBP negative regulation of immune responses; Term 3: GOBP negative regulation of immune system process; Term 4: GOBP negative regulation of T cell differentiation; Term 5: GOBP negative regulation of T cell mediated immunity; Term 6: GOBP negative regulation of T cell proliferation; Spearman analysis). **E** GSEA analysis revealing a significant close correlation of SASP Score elevation and macrophage activation involved in immune response (CMU IDH wt GBM *n* = 26; TCGA IDH wt glioma *n* = 197). **F** The correlation analysis of SASP Score and monocytic lineage based on MCP counter method, as well as that of SASP Score and macrophages based on ssGSEA method, according to indicated glioma datasets (CMU IDH wt GBM *n* = 26; TCGA IDH wt glioma *n* = 197). (ns not significant, * *P* < 0.05, ** *P* < 0.01, *** *P* < 0.001).

Considering that SASP contributes to the constitution of TME by impacting immune cell infiltration [41], we sought to examine whether the SASP Score could be employed to evaluate dysregulated immune responses in IDH wt glioma. Enrichment analysis of genes with a positive correlation coefficient > 0.6 with SASP Score elevation in IDH wt glioma disclosed a consistent enrichment in immune-related BP and KEGG pathways (Table S8) (TCGA, CGGA325, CGGA693, and Gravendeel datasets; Fig. S6A, B). Cancer hallmark analysis with GSEA suggested the enrichment of tumorigenesis-associated pathways and immune-related pathways in samples with SASP Score elevation, such as hallmark of angiogenesis, apoptosis and epithelial-mesenchymal transition, and inflammatory, interferon α, and interferon γ responses (Fig. S7A). Additionally, in terms of immune infiltration and TME analysis, an increased SASP score showed a significant positive correlation with Immune, Stromal, and ESTIMATE scores, while exhibiting a negative correlation with tumor purity (Fig. 3A, B). These results support the association of higher SASP Score with more complex TME constitution in IDH wt glioma [46].

Then, we employed TCGA IDH wt glioma dataset to analyze the anti-cancer immune status of IDH wt glioma by exploring the seven-step cancer immune cycle in TME [29]. IDH wt glioma samples with high SASP Score exhibited enhanced activities in Step 1 (The release of cancer cell antigens), Step 2 (Presentation of tumor antigens), and Step 4 (Transport of immune cells to the tumor). IDH wt glioma samples with low SASP Score had increased activities in Step 3 (The initiation and activation of anti-cancer immunity) and Step 6 (Recognition of cancer cells by T cells) (Fig. S8A and Table S9). These results implied that although SASP activation contributes to the initiation and processing phases of the immune responses, the activation of critical immune responses and recognition of cancer cells by T cells at these phases are inhibited, and the enrichment of SASP Score is still detrimental to the effective anti-tumor immune response. Moreover, GSEA analysis of IDH wt glioma showed that antitumor immune function was suppressed in high SASP Score group (Figs. 3C and S8B, and Table S10). Notably, IDH wt glioma samples with elevated SASP Score exhibits higher expression of immune checkpoint molecules, either co-stimulatory or co-suppressive, compared to samples with low SASP Score (Fig. S8C and Table S11). This may be explained by the “immune tidal model theory”. It has been described that co-stimulatory and co-suppressive immune checkpoints determine the direction and extent of specific immune responses [47]. While co-stimulatory immune checkpoints promote immune responses, secondary co-inhibitory immune checkpoints act as effective negative feedback signals for co-stimulatory factors, and these two roles are highly diverse in monitoring and regulating immune responses, thus potentially contributing to the composition of the immunosuppressive TME. The correlation analysis of three immunosuppressive cell components (Macrophages, neutrophils, and Tregs) and immune suppression pathways in IDH wt glioma disclosed that macrophages were identified to be positively correlated with dampened antitumor immunity in IDH wt glioma (Figs. 3D and S9A and Table S12). The Step 4 analysis of immune cell proportions infiltrating in cancer revealed that SASP activation was positively correlated with the infiltration of macrophages and T cell helper cells, but negatively correlated with the recruitment of CD4 + T cells and CD8 + T cells in IDH wt glioma (Fig. S8A and Table S13). GSEA analysis also supported that SASP was closely related to the activation of macrophages in IDH wt glioma (Figs. 3E and S9B). The analysis with Xcell algorithm indicated more M2 TAMs infiltration in high SASP scoring samples of IDH wt glioma (Fig. S9C) [31]. Besides, the analysis with MCP counter and ssGSEA algorithm indicated the positive correlation between SASP Score elevation and the infiltration of monocytic lineage cells, especially macrophages, in IDH wt glioma (Figs. 3F and S9D) [15, 32, 33]. Collectively, these results supported a positive association of SASP Score elevation to dysregulated immune responses and TAMs infiltration in IDH wt glioma, suggesting an unfavorable role of SASP in anti-tumor immunity within TME of IDH wt glioma.

### Malignant cells and TAMs are the two most abundant cell populations exhibiting SASP in GBM TME

**Fig. 4:**
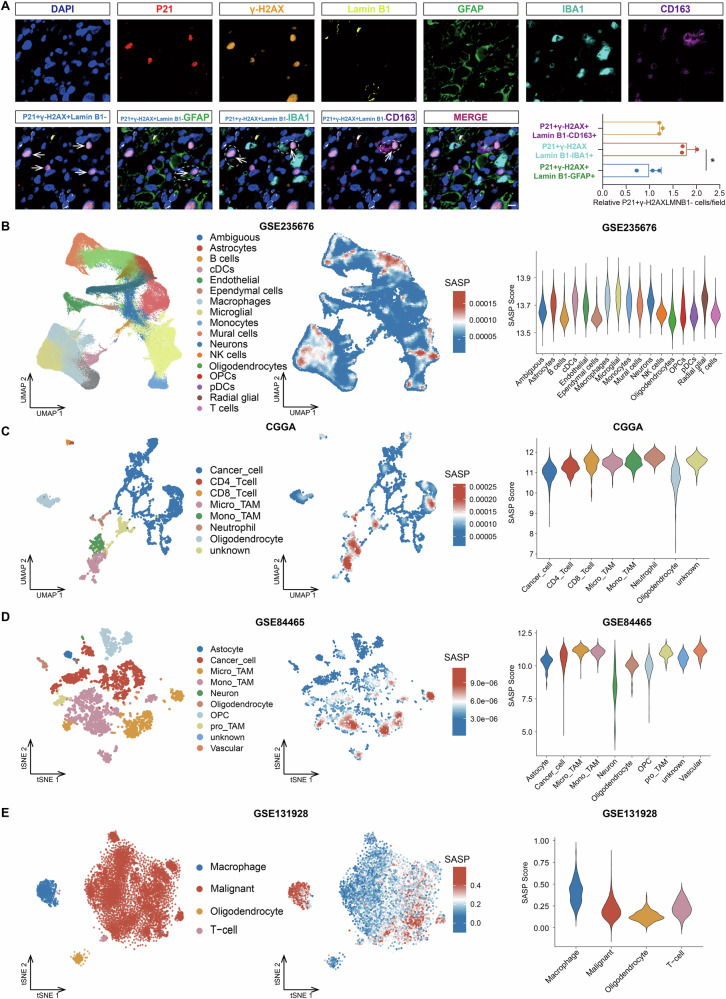
Malignant cells and TAMs present elevated SASP Score in GBM TME. **A** Representative immunofluorescence images of clinical GBM samples. Tumor staining shows the distributions of DAPI (Dark blue), P21 (Red), γ-H2AX (Orange), Lamin B1 (Yellow), GFAP (Green), IBA1 (Cyan), and CD163 (Purple). Images are representative of three images for individual patients. Scale bar, 10 μm. **B–E** The SASP Score analysis of cell components in indicated GBM single cell RNA-seq datasets (**B** GSE235676 *n* = 149048; **C** CGGA *n* = 4193; **D** GSE84465 *n* = 3571; **E** GSE131928 *n* = 7930; Left and middle: The tSNE/UMAP plot (Left) and corresponding SASP Score profile (Middle) in indicated cell populations according to indicated single-cell GBM dataset; Right: Enrichment of SASP Score in the indicated cell types from indicated single-cell GBM datasets).

To further investigate the senescent cell populations in GBM, we performed multiple immunofluorescence (IF) staining analysis on clinical GBM samples. With the employment of markers for senescence cell (P21, γ-H2AX, and Lamin B1), GBM cells (GFAP), Macrophages (IBA1), and M2 macrophages (CD163), we observed that there were significantly more senescent TAMs (detected by P21, γ-H2AX and IBA1 positive, and Lamin B1 negative) than senescent GBM cells (P21, γ-H2AX, and GFAP positive, and Lamin B1 negative) in GBM tumors, and that there was a higher infiltration of M2 TAMs (CD163 positive) in GBM (Fig. 4A). We next sought to delineate the major cell components presenting SASP in GBM TME. We firstly employed a scRNA-seq GBM cohort (GSE235676) to examine the cell components with elevated SASP Score in GBM TME. This analysis revealed that myeloid cells, particularly macrophages, were the most abundant populations with heightened SASP scores in the TME (Fig. 4B). Quantitative analysis further disclosed that TAMs were the cell component with the highest SASP Score in GBM (Fig. 4B). The validation in another three scRNA-seq GBM datasets (CGGA, GSE84465, and GSE131928) demonstrated malignant cells and TAMs, as well as T cells, were the cell populations with elevated SASP Score (Fig. 4C–E). Interestingly, the median SASP Score of malignant cells is significantly lower than that of TAMs (Fig. 4C–E). This result may be attributed to the heterogenicity of GBM cells. Together, these findings suggested that TAMs and malignant cells were the two most abundant cell populations exhibiting SASP in GBM.

### Pharmacological screening based on SASP Score identifies GDC-0879 as a potent SASP inhibitor

**Fig. 5:**
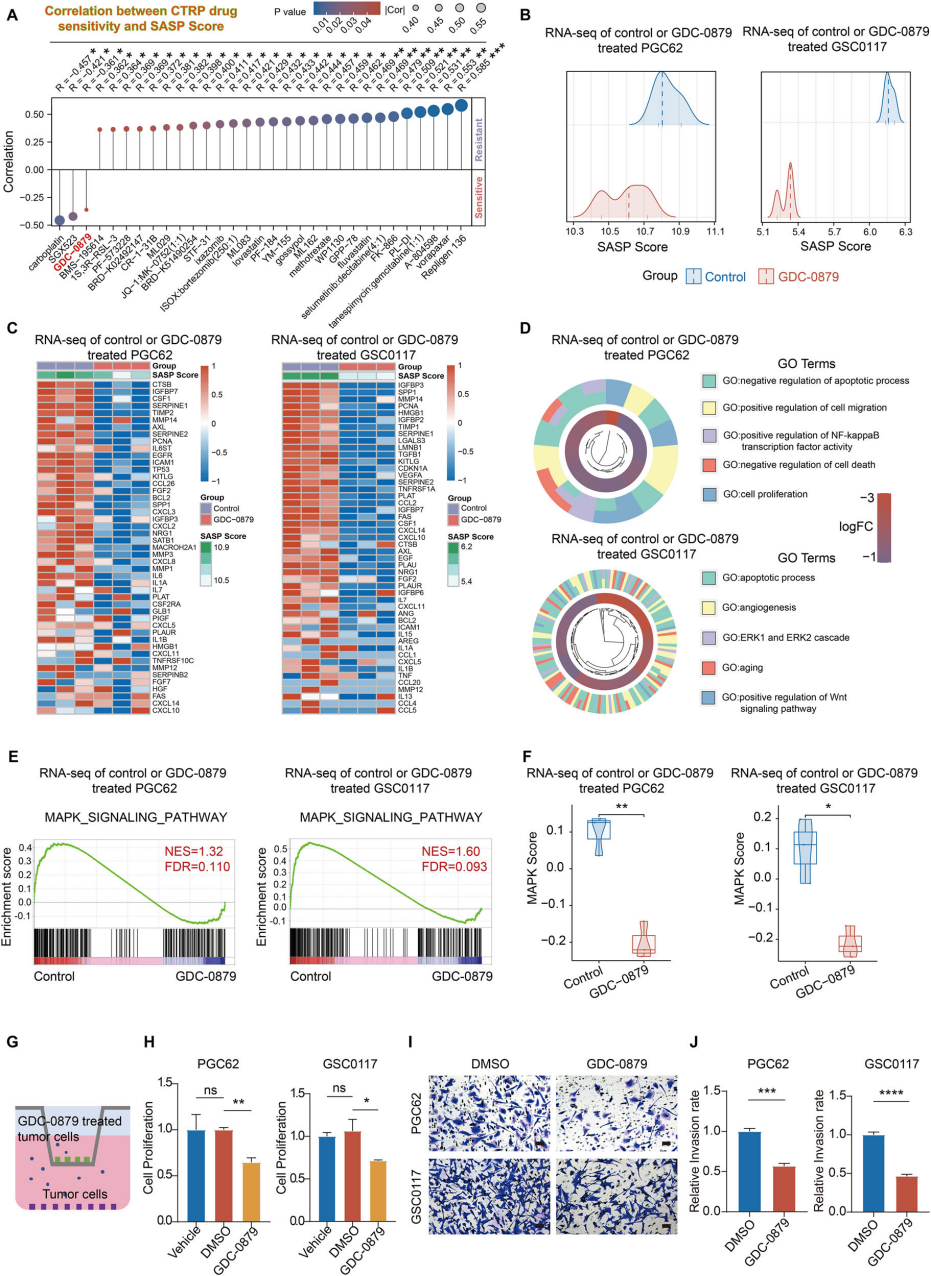
Screening of small molecules identifies GDC-0879 as a potential SASP inhibitor effectively reducing GBM cell proliferation in vitro. **A** Small molecule screening implicating GDC-0879 as a potential SASP inhibitor in glioma cells (The screening data including 583 compounds and 30 glioma cell lines were obtained from The Cancer Therapeutics Response Portal (CTRP, http://www.broadinstitute.org/ctrp) and glioma cell line information was extracted from the Cancer Cell Line Encyclopedia (CCLE) project; AUC was used to represent drug response, in which a small value indicates sensitive in contrast to a large value indicating resistant. Spearman analysis of CTRP drug sensitivity (AUC values) and SASP Scores was calculated to rank the small molecular drug candidates. **B** The analysis of PGC62 (Left) and GSC0117 (Right) samples treated with or without GDC-0879 (10 µM treatment for 48 h), indicating significantly reduced SASP Score accompanied with GDC-0879 administration (*n* = 3). **C** Heatmap analysis showing that reduced SASP Score indicated restrained expression of SASP-associated genes. **D** GO analysis of RNA-seq data obtained from indicated control and GDC-0879 treated GBM cell samples (Left: PGC62, right: GSC0117). **E** GSEA analysis showing that GDC-0879 treatment of GBM cells significantly reduced the enrichment of the gene set associated with the activation of MAPK signaling pathway. **F** The comparison analysis of MAPK scores according to RNA-seq data obtained from indicated control and GDC-0879 treated GBM cell samples (Left: PGC62, right: GSC0117; *n* = 3, *t*-test). **G** The schematic graph showing the co-culture method of GBM cells with GDC-0879 treatment (Upper chamber) and untreated GBM cells (Lower chamber). **H** MTS assays of indicated GBM cells with or without GDC-0879 treatment (Vehicle: PGC62 or GSC0117 alone; DMSO: PGC62 or GSC0117 cocultured with DMSO pretreated PGC62 or GSC0117; GDC-0879: PGC62 or GSC0117 cocultured with GDC-0879 pretreated PGC62 or GSC0117; *n* = 3, one-way ANOVA). **I**, **J** Representative images (**I**) and analysis (**J**) of Transwell invasion assays of indicated GBM cell samples (*n* = 3, *t*-test). Scale bar, 50 μm. (ns not significant, * *P* < 0.05, ** *P* < 0.01, *** *P* < 0.001, **** *P* < 0.0001).

Considering the involvement of SASP activation indicated by the elevation of established SASP score in the malignant progression of IDH wt glioma, we next sought to screen the potential inhibitor of SASP with the SASP Score, which may help the development of strategy improving the therapeutic response of glioma. To better elucidate the therapeutic opportunities and identify the small molecular candidates targeting SASP in glioma, we interrogated drug screening data from Cancer Therapeutic Response Portal (CTRP, http://www.broadinstitute.org/ctrp) and CCLE dataset. The 538 drug candidates included in the screening were filtered by the correlation analysis based on delineating the sensitivity to the drug candidates associated with elevated SASP Scores in 30 glioma cell lines (AUC values, the area under the dose response curve, and smaller AUC values indicate greater sensitivity to the drug candidate) [20, 22]. We identified 31 candidates significantly associated with elevated SASP score (*P* < 0.05). Notably, only three candidates (Carboplatin, SGX523, and GDC-0879) were negatively correlated with the elevation of established SASP score among 538 small molecules (Fig. 5A and Table S14). This indicates that carboplatin, SGX523 and GDC-0879 restrain the elevation of SASP Score and may serve as potential SASP inhibitors in glioma. Considering the side effects of carboplatin on bone marrow, liver, and renal functions, as well as the previously reported nephrotoxicity of SGX523 leading to its failure in clinical trials and the association of SGX523 metabolites with clinically observed obstructive nephropathy [48], we selected GDC-0879 as the candidate for further investigation (Fig. 5A and Table S14).

Indeed, GDC-0879 exhibited a smaller and significant *R* value than well-known SASP inhibitors, Epigallocatechin Gallate and Ruxolitinib (Fig. S10A). According to in vitro IC50 analysis with PGC62 and GSC0117, GDC-0879 has the smallest IC50 (PGC62: 11.76 μM and GSC0117: 13.56 μM) than Epigallocatechin Gallate (PGC62: 27.83 μM and GSC0117: 29.93 μM), Ruxolitinib (PGC62: 44.19 μM and GSC0117: 42.23 μM), and Rapamycin (PGC62: 14.75 μM and GSC0117: 18.95 μM, another SASP inhibitor) (Fig. S10B, C) [49–51]. Additionally, doxorubicin-induced senescent GBM cells were more sensitive to GDC-0879 treatment compared to control cells (Fig. S10D, E). After treating two doxorubicin-induced senescence GBM cells (PGC62 and GSC0117) with 10 μM GDC-0879, we observed a significant decrease in senescent SA-β-gal staining and a significant downregulation of P21 mRNA expression (Fig. S10F–I). Additionally, RT-qPCR demonstrated that GDC-0879 treatment (10 μM) not only induced a significant reduction in mRNA expression of the SASP-related genes in senescent GBM cells (IL-6, IL1α, IL1β, TNFα, MMP3, CXCL10, and CCL2; Fig. S10H, I), but also exhibited a more efficient inhibition on mRNA expression of SASP cytokines (IL1α, IL1β, TNFα, and CXCL10) than Epigallocatechin Gallate, Ruxolitinib, and Rapamycin at their IC50 concentration (Fig. S10J, K). This indicated a potentially stronger inhibitory effect of GDC-0879 on SASP activation than these SASP inhibitors. The subsequent RNA-seq detection disclosed a decreased SASP Score induced by GDC-0879 treatment (Fig. 5B). Heatmap analysis showed that reduced SASP Score efficiently reflected reduced SASP-associated gene expression in GBM cells (Fig. 5C). Then, to dissect the potential mechanism related to GDC-0879 administration in GBM cells, we performed RNA-seq of primary GBM cell samples with GDC-0879 or control treatment. GO BP and KEGG analysis showed that GDC-0879 treatment mainly induced the enrichment in the BPs associated with apoptotic process and oncogenic signaling pathways like MAPK signaling pathway, Wnt signaling pathway, and NF-κB signaling pathway. (Figs. 5D, E and S11A, B). Then, GSEA and ten classical oncogenic signaling pathway [52] score analysis constructed by “GSVA” R package together disclosed that GDC-0879 treatment significantly inhibited the activation of MAPK and Hippo signaling pathways in GBM cells (Table S14). Since the analysis by GO, KEGG, and GSEA all supported GDC-0879 restraining MAPK signaling and GDC-0879 has been indicated to target this pathway, we chose MAPK for further investigation (Fig. 5D, E and Table S14). We constructed a MAPK score with MAPK signaling pathway gene set downloaded from the Molecular Signatures Database [42] for evaluating the activation of MAPK pathway by GSVA, which demonstrated a significantly reduced MAPK score accompanied with GDC-0879 treatment (Fig. 5F). Immunoblotting of MAPK signaling targets, including BRAF, ERK, p-ERK, P38, p-P38, JNK, and p-JNK in PGC62 and GSC0117 GBM cells with control, doxorubicin, or GDC-0879 treatment after doxorubicin induction further supported this observation (Fig. S11C). These data support GDC-0879 functioning through MAPK pathway, while targeting SASP in GBM cells. Given the well-established role of aberrant activated MAPK signaling in mediating malignant cell proliferation, GDC-0879 may inhibit GBM cell growth through restraining MAPK signaling activation. Moreover, the data obtained from co-culture experiment of untreated GBM cells and Doxorubicin-induced senescence GBM cells with GDC-0879 pretreatment demonstrated that the proliferation abilities and invasion of untreated GBM cells induced by senescence GBM cells were significantly reduced by GDC-0879 pretreatment (Fig. 5G–J). Meanwhile, GDC-0879 treatment at 10 μM significantly increased the proportion of apoptotic cells in senescence GBM cells samples induced by 200 and 100 nM Doxorubicin, compared to non-senescence GBM cells (as shown in Fig. S11D, senescence in the two primary GBM cell lines induced by 100 nM Doxorubicin treatment was confirmed by SA-β-gal staining) (Fig. S11E, F). Together, these results indicated GDC-0879 as a small molecule with SASP inhibition potential.

### GDC-0879 significantly restrains the enhanced proliferation and invasion of GBM cells induced by senescence TAMs presenting SASP

**Fig. 6:**
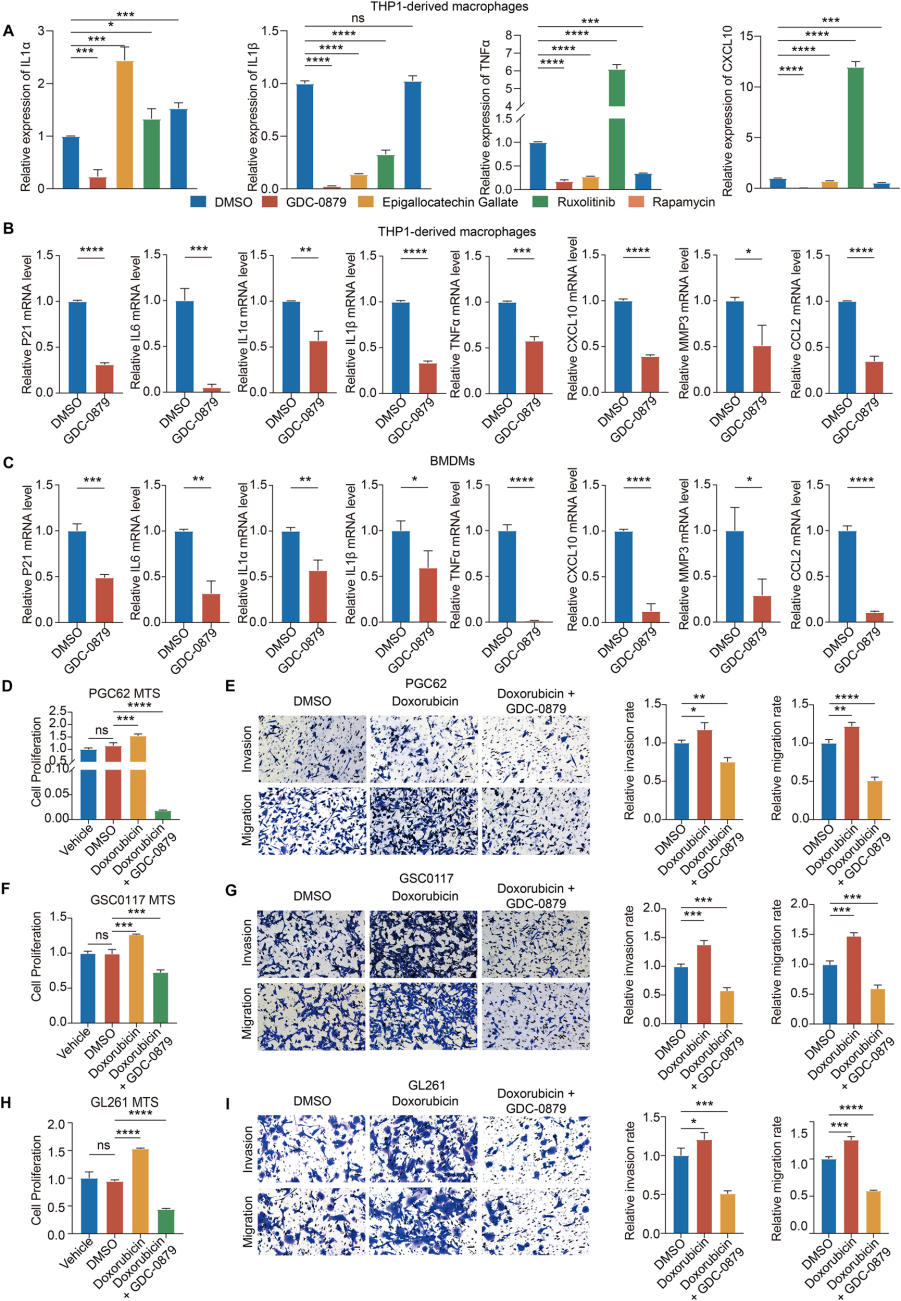
GDC-0879 treatment efficiently reduces macrophage M2 polarization. **A** qPCR analysis of indicated marker (IL1α, IL1β, TNF-α, and CXCL10) mRNA in THP1-derived senescent macrophage samples induced by Doxorubicin and then treated with GDC-0879, Epigallocatechin Gallate, Ruxolitinib, and Rapamycin at their IC50 concentration (*n* = 3, *t*-test). **B**, **C** RT-qPCR analysis of indicated SASP marker genes in THP-1 (**B**) and mouse BMDMs (**C**) derived senescent macrophages induced by Doxorubicin (200 nM) and then with or without GDC-0879 treatment (10 μM) (*n* = 3, *t*-test). **D** MTS assays of PGC62 cell samples cocultured with control or Doxorubicin pretreated THP-1 derived macrophages with or without GDC-0879 treatment (Vehicle: PGC62 alone; DMSO: PGC62 cocultured with DMSO pretreated THP-1 derived macrophages; Doxorubicin: PGC62 cells cocultured with 200 nM Doxorubicin pretreated THP-1 derived macrophages; GDC-0879: PGC62 cocultured with THP-1 derived macrophages pretreated with 200 nM Doxorubicin and then treated with 10 μM GDC-0879; *n* = 3, one-way ANOVA). **E** Representative images (Left) and analysis (Right) of Transwell invasion (Upper) and migration (Lower) assays of PGC62 cell samples with the coculture of control or Doxorubicin (200 nM) pretreated THP-1 derived macrophages with or without GDC-0879 treatment (10 μM) (*n* = 3, one-way ANOVA). Scale bar, 50 μm. **F** MTS assays of indicated GSC0117 cocultured with control (DMSO) or Doxorubicin (200 nM) pretreated THP-1 derived macrophages with or without GDC-0879 treatment (10 μM) (Vehicle: GSC0117 alone; DMSO: GSC0117 cocultured with DMSO pretreated THP-1 derived macrophages; Doxorubicin: GSC0117 cocultured with Doxorubicin pretreated THP-1 derived macrophages; GDC-0879: GSC0117 cocultured with THP-1 derived macrophages pretreated with 200 nM Doxorubicin and then treated with 10 μM GDC-0879; *n* = 3, one-way ANOVA). **G** Representative images (Left) and analysis (Right) of Transwell invasion (Upper) and migration (Lower) assays of GSC0117 cell samples with the coculture of control or Doxorubicin (200 nM) pretreated THP-1 derived macrophages with or without GDC-0879 treatment (10 μM) (*n* = 3, one-way ANOVA). Scale bar, 50 μm. **H** MTS assays of GL261 cells cocultured with mouse BMDMs with indicated pre-treatment (Vehicle: GL261 alone; DMSO: GL261 cocultured with mouse BMDMs pretreated with DMSO; Doxorubicin: GL261 cocultured with mouse BMDMs pretreated with 200 nM Doxorubicin; GDC-0879: GL261 cocultured with mouse BMDMs pretreated with 200 nM Doxorubicin and then treated with 10 μM GDC-0879; *n* = 3, one-way ANOVA). **I** Representative images (Left) and analysis (Right) of Transwell invasion (Upper) and migration (Lower) assays of GL261 cells cocultured with control (DMSO) or Doxorubicin (200 nM) pretreated mouse BMDMs with or without GDC-0879 treatment (10 μM), respectively (*n* = 3, one-way ANOVA). Scale bar, 50 μm. (ns not significant, * *P* < 0.05, ** *P* < 0.01, *** *P* < 0.001, **** *P* < 0.0001).

Since TAMs were one of the abundant cell populations with the highest SASP Score in GBM TME, we next employed two widely accepted macrophage models, THP-1 derived macrophages and mouse BMDMs, to examine whether GDC-0879 could efficiently suppress SASP activation in TAMs. The induction of cellular senescence in THP-1 derived macrophages and BMDMs by Doxorubicin (200 nM) was confirmed by SA-β-Gal staining (Fig. S12A, B), which also led to a significant mRNA elevation of SASP genes in these cells (P21, IL6, IL1α, IL1β, TNFα, CXCL10, MMP3, CXCL10, and CCL2; Fig. S12C, D). Similar to the observations of GDC-0879 on SASP of GBM cells (Fig. S10J, K), qPCR revealed that GDC-0879 treatment efficiently inhibited the mRNA expression of SASP cytokines (IL1α, IL1β, TNFα, and CXCL10) in senescence-induced THP-1 derived macrophages than Epigallocatechin Gallate, Ruxolitinib, and Rapamycin at their IC50 concentration (Fig. 6A). Besides, qPCR revealed a significantly reduced mRNA expression of P21, IL6, IL1α, IL1β, TNFα, CXCL10, MMP3, CXCL10, and CCL2 in THP-1 derived macrophages and BMDMs after GDC-0879 treated (Fig. 6B, C). In addition, GDC-0879 treatment significantly decreased CD206 and CD163 mRNA expression in these cell samples, while increasing their CD86 and CD80 mRNA expression. This indicated a potential restraint triggered by GDC-0879 treatment on M2 polarization of TAMs (Fig. S12E). Similarly, the enhanced proliferation abilities of GBM cells induced by the co-culture of Doxorubicin-induced senescence THP-1 derived macrophages and BMDMs were efficiently decreased by 10 μM GDC-0879 treatment (Fig. 6D, F, and H). Moreover, the co-culture of GBM cells with macrophages pre-treated with Doxorubicin (200 nM) or GDC-0879 (10 μM) showed that Doxorubicin-induced senescence THP-1 derived macrophages and BMDMs significantly enhanced the migration and invasion of GBM cells, which could be reversed by GDC-0879 (Fig. 6E, G, and I and S12F). Thus, it is reasonable to conclude that GDC-0879 not only significantly reduced the M2 polarization of TAMs, but also limited the promotive effect of SASP factors from TAMs on GBM cell migration, invasion, and proliferation in vitro.

### GDC-0879 significantly reduces the tumorigenicity of GBM cells in vivo and improves their response to PD1 blockade

**Fig. 7:**
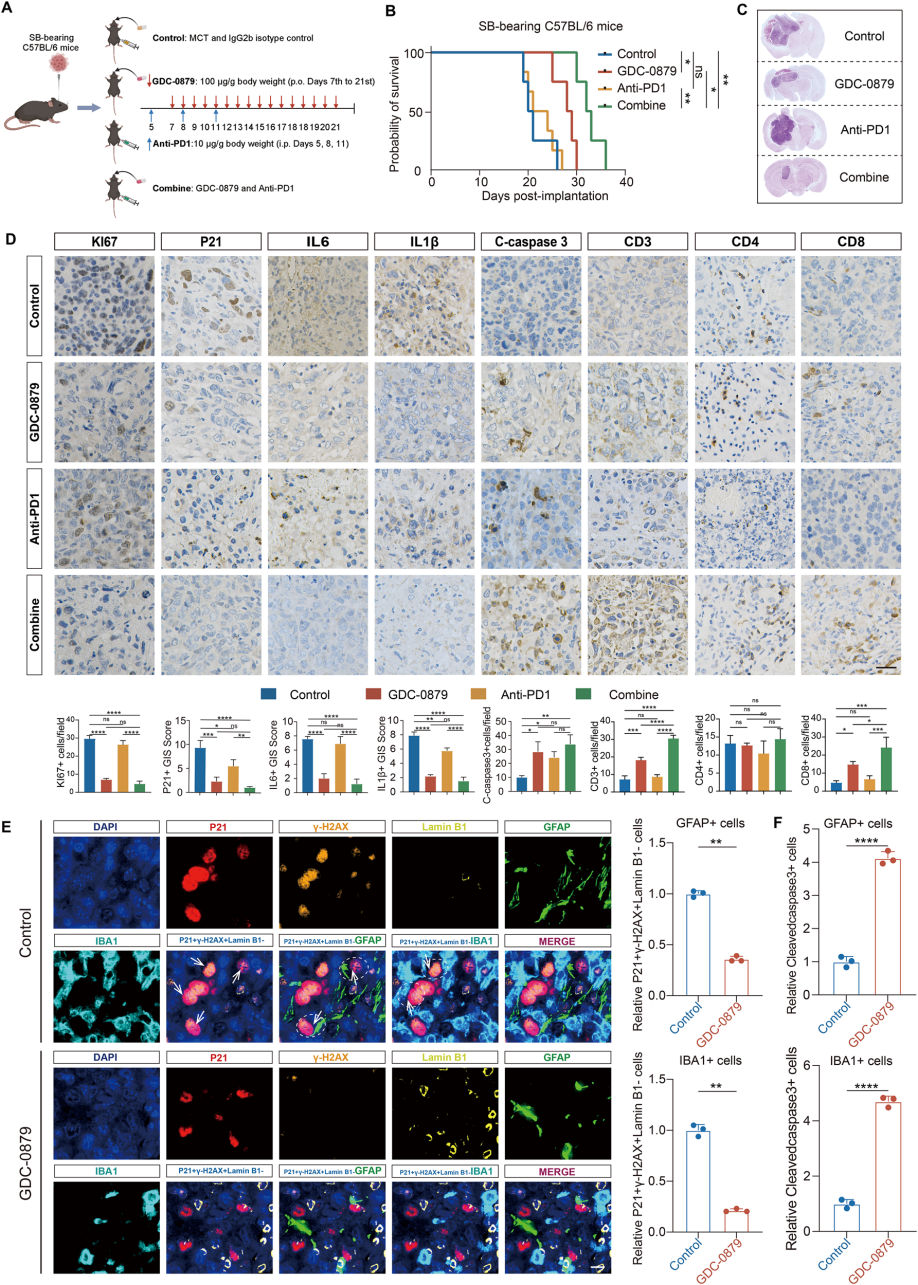
GDC-0879 treatment significantly reduces GBM cell tumorigenicity and improves their response to PD1 blockade. **A** The schematic graph showing the administration method of indicated treatments in SB mouse GBM cell derived intracranial tumor-bearing mouse models. Figure 7A was Created in BioRender. **B** Survival plots of mice intracranially transplanted with SB GBM cells and then receiving indicated treatment shown in Fig. 7A (Control: receiving a single oral dose of 0.5% methylcellulose/0.2% Tween 80 (MCT) and IgG2b isotype control; GDC-0879: GDC-0879 (100 μg/g body weight) in MCT was orally administered by gavage daily from 7th to 21st day; Anti-PD1: Anti-PD1 antibody (10 μg/g body weight) was injected intraperitoneally on the 5th, 8th, and 11th day after the intracranial implantation of SB GBM cells; Combined treatment: treated with GDC-0879 and anti-PD1; Log-rank test, *n* = 6). **C** Representative H&E staining images of brain sections from indicated mice groups on day 21 after intracranial transplantation of SB mouse GBM cells. **D** Representative immunohistochemical staining images and analysis of P21, IL6, IL1β, C-caspase-3 (cleaved Caspase-3), CD3, CD4, and CD8 in SB intracranial xenograft sections from indicated mice groups (*n* = 3, one-way ANOVA). Scale bar, 25 μm. **E** Representative immunofluorescence images of DAPI (Dark blue), P21 (Red), γ-H2AX (Orange), Lamin B1 (Yellow), GFAP (Green), and IBA1 (Cyan) in SB intracranial xenograft sections from indicated mice groups (*n* = 3, *t*-test). Scale bar, 10 μm. **F** The ratio of cells with positive expression of GFAP and cleaved caspase 3, and IBA1 and cleaved caspase 3 treated by GDC-0879 treatment in SB intracranial xenograft sections from indicated mice groups (*n* = 3, *t*-test). (ns not significant, * *P* < 0.05, ** *P* < 0.01, *** *P* < 0.001, **** *P* < 0.0001).

Since we identified the involvement of SASP in mediating the dysregulated immune responses and contributing to the immunosuppressive TME of GBM, we next sought to examine whether GDC-0879 could restrain GBM SASP in vivo and sensitize them to immunotherapy like ICB. Somatic mutations have been implicated as a predictor of cancer response to immunotherapy and tumors with high mutation burden are considered to have higher immunogenicity and therefore may actively respond to ICB, due to their increased neoantigen burden [53]. After stratified TCGA IDH wt glioma sample with the median value of SASP Score, in terms of somatic mutations, the mutation rate was 78.72% in low SASP Score group, higher than that in high SASP Score group (67.37%) (Fig. S13A, B). This suggested that immunotherapy may bring more benefits to patients with low SASP Score. Then, the immunotherapy response and survival analysis with three cancer transcriptomic cohorts receiving ICB, including melanoma (Fig. S13C, GSE78220, *n* = 28, with anti-PD1; Fig. S13D, Van-allen, *n* = 42, with anti-CTLA4) and metastatic urothelial cancer (Fig. S13E, IMvigor210, *n* = 348, with anti-PD-L1) demonstrated that the SASP Score may efficiently stratify the patients’ response to anti-PD1 (GSE78220) instead of other two ICB strategy [9, 54]. Similarly, SASP Score grouping distinguished patients’ survival in GSE78220 (with anti-PD1, Fig. S13F), instead of the other two cohorts treated with anti-PD-L1 and anti-CTLA4, respectively (Fig. S13G, H). The analysis of GBM treated with anti-PD1 (GSE235676) based on present SASP Score indicated that SASP Score was more enriched in the cell samples from GBM patients who did not respond to PD1 blockade (Fig. S14A, B) [11]. Therefore, we sought to examine whether GDC-0879 treatment could improve GBM response to PD1 blockade, while clarifying the efficacy of GDC-0879 strategy on GBM progression in vivo. Intracranial SB mGSC transplantation C57BL/6 immune competent mice model was employed to conduct GBM orthotopic xenograft experiments. The administration of anti-PD1 in tumor-bearing mice based on the method described by previous studies [16, 55, 56]. The application of GDC-0879 also followed the strategy recommended by published reports [57, 58]. (Fig. 7A). Indeed, GDC-0879 monotherapy was effective in prolonging the survival of mice, while anti-PD1 therapy didn’t bring significant survival benefit, and the combination therapy brought a significantly better survival benefit than GDC-0879 alone (Fig. 7B). GDC-0879 not only limited the proliferation of SB mouse GBM cells in vivo, but also significantly improved the effectiveness of anti-PD1 immunotherapy (Fig. 7B, C). Consistent with the results obtained from in vitro assays (Fig. S10H, I), IHC staining disclosed a significantly suppressed expression of senescence marker P21 and SASP cytokines like IL6 and IL1β in tumor samples, regardless of GDC-0879 mono- or combined therapy with anti-PD1 (Fig. 7D). This suggested that GDC-0879 significantly reduces senescence cells as well as SASP factor secretion in GBM. Both of GDC-0879 mono- and combined therapy with anti-PD1 significantly increased the IHC staining intensity of cleaved-caspase 3 in these samples, indicating the increased apoptotic cell ratio induced by GDC-0879 treatment (Fig. 7D). IF analysis further disclosed that GDC-0879 treatment reduced the number of senescent cells (identified with γ-H2AX, and P21 positive expression, and Lamin B1 negative expression). Moreover, both senescence GBM cells (identified with GFAP, γ-H2AX, and P21 positive, and LaminB1 negative expression) and TAMs (identified with IBA1, γ-H2AX, and P21 positive, and Lamin B1 negative expression) were reduced by GDC-0879 (Fig. 7E). The combination of GDC-0879 and PD1 blockade led to an increase in CD3+ and CD8+ lymphocytes (Fig. 7D), which suggested that GDC-0879 and combination treatment of GDC-0879 and anti-PD1 improved T cell antitumor immunity.

GL261-bearing C57BL/6 immune competent mice model was also employed to conduct GBM orthotopic xenograft experiments, and treated mice with anti-PD1 antibody, GDC-0879, and the combination of these two strategies, respectively (Fig. S15A). Although both the administration of anti-PD1 and GDC-0879 monotherapy effectively prolonged mice survival, the combination strategy brought a significantly better survival benefit than GDC-0879 and anti-PD1 alone (Fig. S15B). GDC-0879 treatment restrained the orthotopic proliferation of GL261 cells (Fig. S15C) and reduced the IHC staining intensity of Ki67 in orthotopic xenografts (Fig. S15D). This reflected its capability to reduce GBM cell tumorigenicity in vivo, while its combination with anti-PD1 treatment further strengthened this effect (Fig. S15D). IHC staining disclosed a significantly suppressed expression of senescence marker P21 and SASP cytokines like IL6 and IL1β in tumor samples, regardless of GDC-0879 mono- or combined therapy with anti-PD1 (Fig. S15D). Both of GDC-0879 mono- and combined therapy with anti-PD1 significantly increased the IHC staining intensity of cleaved-caspase 3 in these samples, indicating the increased apoptotic cell ratio induced by GDC-0879 treatment (Fig. S15D). IF analysis disclosed that GDC-0879 treatment reduced the number of senescent cells (identified with γ-H2AX, and P21 positive expression, and Lamin B1 negative expression). Moreover, both senescent glioma cells (identified with GFAP, γ-H2AX, and P21 positive, and Lamin B1 negative expression) and TAMs (identified with IBA1, γ-H2AX, and P21 positive, and Lamin B1 negative expression) were reduced by GDC-0879 (Fig. S15E). The combination of GDC-0879 and PD1 blockade led to an increase in CD3+ lymphocytes (Fig. S15D), and GDC-0879 and combination treatment group samples demonstrated an enrichment of CD4+ and CD8 + T cells, which suggested that GDC-0879 and combination treatment of GDC-0879 and anti-PD1 improved T cell antitumor immunity (Fig. S15D). IF analysis disclosed that GDC-0879 treatment increases apoptosis ratios of GBM cells and macrophages in SB and GL261 mice orthotopic xenograft (Figs. 7F and S16A–C). Meanwhile, the detection of reduced CD206+ and elevated CD86+ cell ratio in GDC-0879 treatment group suggested that GDC-0879 reduced the infiltration of M2-like TAMs. This trend was further strengthened by the combination strategy with anti-PD1 (Fig. S17A, B). FACS analysis revealed that combined treatment had significantly improved MHCII+ cell infiltration, while CD206+ cell infiltration was decreased, indicating that GDC-0879 suppressed the infiltration of M2-like TAMs (Fig. S17C, D). Moreover, the combination of GDC-0879 and anti-PD1 didn’t bring significant liver and renal toxicity in tumor-bearing mice (Fig. S18A, B), which supports the potential clinical translation feasibility of this treatment strategy. In addition, IHC analysis of brain sections of GL-261 and SB mouse GBM cell-derived intracranial tumor-bearing mice demonstrated that GDC-0879 treatment significantly reduced the staining intensities of the MAPK signaling targets, including BRAF, ERK, p-ERK, P38, p-P38, JNK, and p-JNK (Fig. S19A, B), supporting the potential role of GDC-0879 functioning through MAPK pathway, while targeting SASP in GBM. Besides, Tumor Treating Field (TTF) treatment is a recent progress in GBM therapeutics functioning through inhibiting the mitosis of GBM cells and inducing senescence in these cells [59]. The analysis of RNA-seq data obtained from a recent study disclosed a significant elevation of SASP score in GBM samples with TTF treatment in comparison with control samples (Fig. S20A). Together, these data support GDC-0879 as a small molecule targeting SASP in GBM, which brings survival benefits in mice preclinical models and improves their responses to anti-PD1.

## Discussion

While senescence induces the proliferation arrest of cancer cells and non-malignant cells in the TME, it plays ambivalent roles in oncogenesis and may contribute to cancer progression through the intercellular communication mechanism, SASP [3]. In present study, we generate a SASP gene set and establish an SASP Score with ssGSEA method, which effectively reflects SASP activation status in glioma. Then, through an integrated transcriptomic analysis, we find that IDH wt glioma exhibits an elevated SASP Score than IDH mutant glioma, which serves as an important feature for the progression of IDH wt glioma. Screening based on the SASP Score identify a small molecular candidate for SASP inhibition, GDC-0879, which administration decreases the tumorigenicity of GBM cells and improves their responses to anti-PD1.

A recent study defined a GBM senescence signature by calculating a ssGSEA senescent Z-score corresponding to 31 DEGs, and single-cell data analysis showed that mouse senescence signature was conserved in patient GBM [2]. The 91 gene candidates included in our gene panel could be classified into the following two broad categories: senescence markers and SASP factor-encoding genes. Considering the context-dependent nature of senescence, the senescence markers included in our panel are not only from the gene encoding different hallmarks of senescence, but also cover the senescence markers more common in CNS. The SASP factor genes included in our gene set cover the genes encoding chemokines, growth factors and regulators, interleukins, proteases and regulators, soluble or shed receptors or ligands, inflammatory factors, and classical senescence markers (Table S1). This SASP scoring method could be applied for bulk and scRNA-seq data analysis to identify cells with high levels of SASP factor encoding gene expression. Moreover, our gene set comprised the genes encoding the regulators of senescence characteristics, especially a focused overview of the most prominent SASP features contributing to cancer progression, as well as covering the classical senescence markers of CNS, which allows us to construct a SASP scoring model that quantifies the level of senescence cells and SASP activation in glioma and minimized the possibility of confounding with phenotypic features other than non-senescence [2, 60–62]. The effectiveness of our SASP gene set was validated in our own CMU GBM dataset, as well as in other four large-size glioma datasets, which supports the potential of our work in assessing GBM SASP status. Indeed, our SASP Score has the potential serving as a more applicable quantification and evaluation system for SASP in glioma compared to previous senescence models constructed in glioma [35–38] through combining the senescence marker genes with SASP factor encoding genes in Table S1. Meanwhile, the incorporation of multiple senescence markers in current gene panel would be conducive for overcoming the limitation of single senescence marker like CDKN2A, which presents insufficient expression abundance at transcriptomic level, especially in scRNA-seq [34]. The integration of genes encoding classical senescence markers, especially markers more common in CNS, make our panel more suitable for the detection of SASP in glioma, instead of other inflammation responses. For example, in the comparison of our SASP Score with 4 other senescence signatures [35–38], only our SASP Score consistently shows significant upregulation in the 9 senescence induction models (Fig. S1A), which supports the validity of our signature in SASP evaluation. Another interesting finding is that we observed that primary GBM cell PGC62 exhibits a higher SASP Score compared GSC0117 cultured in serum-free medium. This may be due to the genetic background difference between samples. However, the comparison of paired GSC and serum-induced DGC samples also disclosed a significantly elevated SASP Score in DGC samples than GSC (Data not shown). Considering that GSC presents stronger tumorigenicity than paired serum-induced DGC, it is worthy for further investigation whether there exists differential SASP activation between GSC and DGC population and their roles in facilitating GBM progression. Furthermore, it is worth noting that pro-inflammatory secreted SASP factors also function during inflammatory response. The inflammation characteristics and context-dependent nature of SASP may impact the evaluation fidelity of present SASP panel despite the incorporation of multiple senescence marker encoding genes in current panel reduces the probability of confounding with other inflammatory responses.

Previously, it has been revealed that the constitutions of cancer-infiltrating immune cells and the interaction between these cells and malignant cells varied with the genetic background of cancers and the composition of accompanied SASP [41]. Interestingly, current study identified cancer cells and TAMs as the most abundant cell population presenting SASP in GBM TME. The enrichment of SASP Score in IDH wt glioma may be a reflection of IDH wt glioma harboring more intra-tumoral macrophages in TME compared to IDH mutation tumors with more microglia. scRNA-seq implicates that GBM cell senescence is a heterogeneous phenotype. The enrichment of SASP score in MES-like cellular state of GBM cells. This may reflect the close association of MES identity or cellular state in malignant cells with stress responses. While senescent cancer cells present proliferation arrest, they facilitate the proliferation of non-senescent malignant cells by paracrine signals of SASP. Senescence cells secrete a diversity of bioactive proteins, which contributes to tumorigenesis through the context-dependent mechanisms and may reinforce the senescence program in an autocrine manner.

Another finding in present work is that SASP potentially impacts GBM response to anti-PD1 therapy and GDC-0879 was identified as a small molecular candidate targeting SASP in glioma by SASP inhibitor screening in glioma cells based on current SASP Score. GDC-0879 was primarily reported as a BRAF mutant inhibitor, and may exert opposing cellular context-dependent functions on tumor growth. GDC-0879 showed specific inhibition on cancers with BRAF(V600E) mutation through blocking MAPK signaling, a common oncogenic pathway [52, 63], in contrast to enhancing tumor growth in KRAS mutant and RAS/RAF wild-type tumors like melanoma and lung cancer by exhibiting RAS-dependent activation on RAF-MEK-ERK pathway through direct conformational effects on RAF kinase domain [64]. Additionally, GDC-0879, as a highly selective small molecule inhibitor of RAF, has variable functions on the activation of RAF and MEK in malignant cells [63]. The inhibition of GDC-0879 on MEK restrains the proliferation of cancer cell lines carrying wild-type BRAF with different KRAS status (81% KRAS mutant, 38% KRAS wild-type) [63]. It has been reported that physiological activation of the MAPK pathway is caused by stimulation of ligand-dependent tyrosine kinase transmembrane receptors (RTKs), which homo- or hetero-dimerize leading to the phosphorylation and activation of a downstream cascade involving the RAS, RAF kinase, MEK1/2, and ERK. Activated ERK proteins translocate to the nucleus, where they phosphorylate and regulate multiple transcription factors, modulating downstream target transcription. The activation and transduction of this signaling pathway drive cell proliferation, differentiation, survival, or senescence [65]. Indeed, sustained activation of the RAS/MAPK signaling pathway leads to cell proliferation arrest by affecting p53 and/or p16 activities [66]. After observing the restriction effect of GDC-0879 on the proliferation of two primary GBM cell lines in vitro, we assessed the therapeutic effect of GDC-0879 in vivo. We firstly employed GL261 derived immunocompetent murine orthotopic xenograft model, which is widely used for evaluating immunotherapy efficiency in GBM. The administration of GDC-0879 significantly extended the survival of mouse GL261 GBM preclinical model and improved their response to anti-PD1. Given that GL261 is recognized as a hypermutated GBM model [30], we further utilized SB mouse GBM cells derived murine orthotopic tumor model established by SB transposon technique to examine the therapeutic efficiency of GDC-0879, which is considered to more reliably recapitulate human GBM characteristics compared to GL261 model [31]. Similarly, GDC-0879 treatment brought substantial survival benefit to SB tumor-bearing mice and ameliorated their sensitivities to anti-PD1. To date, the application of GDC-0879 in GBM treatment remains quite limited. In present study, we observed significant activation of MAPK signaling pathway induced by doxorubicin treatment, which could be efficiently inhibited by the application of GDC-0879. This provides a potential explanation for GDC-0879 mediated SASP inhibition in GBM. Since the context-dependent mechanism has been indicated for GDC-0879 application in cancer, the further investigation is required to clarify the direct interacting partners and downstream effectors of GDC-0879 in GBM cells and the cell populations affected by GDC-0879 in GBM TME. We also observed a significant inhibitory effect of GDC-0879 on the activation of HIPPO pathway through the GSEA analysis (Table S15), which is an important aspect for subsequent research. Moreover, the application of GDC-0879 significantly reversed the pro-tumorigenic TAMs to pro-inflammatory phenotype, which indicated its potential to unbound the suppression of TAMs on immunosurveillance in TME and enhanced cytotoxic CD8 + T cell infiltration, which improved cancer response to anti-PD1. Moreover, with the development of neoadjuvant immunotherapy, the importance of time-setting of immune therapeutic strategy is increasingly recognized by researchers. Since GDC-0879 sensitizes GBM to anti-PD1, the optimization of current administration setting on applicating GDC-0879 before anti-PD1 may further sensitize GBM to PD1 blockade, which is worthy further investigation.

TTF treatment, as a strategy recently adopted into the standards of care in GBM therapy, has been reported to induce senescence in GBM cells in mediating its anti-cancer impacts [59]. The analysis of RNA-seq data obtained from a recent report [10] on the potential mechanism of TTF treatment against GBM, which revealed GBM samples with TTF treatment demonstrating an elevation of SASP Score compared to control samples. This supported TTF treatment, inducing GBM senescence, while achieving therapeutic benefits. However, cellular senescence is a complex multifaced biological process. It has a dual effect on cancer progression. Due to the notorious heterogenous nature of GBM, malignant cells exhibit varying response to TTF. While senescence of malignant cells is conducive to the control of tumor progression through inducing proliferation arrest, it may facilitate cancer progression through SASP by affecting the functions and behaviors of TME components, including adjacent malignant cells survival from TTF and immune cells through paracrine effects [2, 3]. The combination of small molecule targeting SASP like GDC-0879 and TTF treatment may have the potential to disrupt the delicate balance elaborately constructed by malignant cells and further bring therapeutic benefit to GBM patients receiving TTF therapy, which is worthy for further investigation.

In summary, we provide a SASP Score with generating a SASP gene panel. The validation of this score in function assays and cancer senescence datasets demonstrates that it could efficiently reflect SASP activation. Additionally, our study highlights that IDH wt glioma exhibits a more active SASP phenotype compared to IDH mutant tumor and GBM exhibits a pro-tumorigenic context-dependent SASP activation, especially in malignant cells and TAMs. The screening with the SASP Score identified GDC-0879 as a small molecular SASP inhibitor improving the efficiency of anti-PD1 therapy in mice GBM preclinical model. The combination of SASP inhibition and anti-PD1 could be taken into account as a potential strategy for future clinical studies.

## Supplementary information

Supplementary figures

Supplementary tables

Original File of Western Blot
