# Supplementary material for: Profiling with senescence-associated secretory phenotype score identifies GDC-0879 as a small molecule sensitizing glioblastoma to anti-PD1
Source: Cell Death Dis. 2025 Aug 9;16(1):602. doi: 10.1038/s41419-025-07915-3 (PMC12334699; doi:10.1038/s41419-025-07915-3)
Supplement: Supplementary file 1 — Supplementary figures [file 41419_2025_7915_MOESM1_ESM.docx]

**Supplementary Figures**

**Profiling with senescence-associated secretory phenotype score identifies GDC-0879 as a small molecule sensitizing glioblastoma to anti-PD1**

Yang Liu ^1, 2^**^#^**, Yuan Feng ^2^**^#^**, Lin Cheng ^1^**^#^**, Yangxi Xu ^3^, Anhua Wu^2, 4^, Peng Cheng^1, 4^

1. Department of Neurosurgery, The First Hospital of China Medical University, Shenyang, Liaoning, China

2. Department of Neurosurgery, Shengjing Hospital of China Medical University, Shenyang, Liaoning, China

3. Department of Neurosurgery, the People’s Hospital of Liaoning Province, Shenyang, Liaoning, China

4. Institute of Health Sciences, China Medical University, Shenyang, Liaoning, China

**# Authors contributed equally to this work.**

**Correspondence Authors:**

Peng Cheng, Department of Neurosurgery, The First Hospital of China Medical University, No.155 Nanjingbei Street, Heping District, Shenyang, Liaoning 110001, China, (chengpeng@cmu.edu.cn; Phone number: +86-024-83283133).

Anhua Wu, Department of Neurosurgery, Shengjing Hospital of China Medical University, No. 36, Sanhao Street, Heping District, Shenyang, Liaoning 110004, China, (ahwu@cmu.edu.cn; Phone number: +86-024-22958989).

**This file includes: Supplementary Figures and Figure legends**

**Figure S1 related to Figure 1.** Comparison of current SASP gene panel with four published senescence gene sets in senescence evaluation according to indicated datasets.

**Figure S2 related to Figure 1.** Elevated SASP Score efficiently represents SASP activation in GBM cells.

**Figure S3 related to Figure 2.** SASP activation is intimately associated with the malignant progression of IDH wt glioma.

**Figure S4 related to Figure 2.** SASP Score elevation is closely correlated with poor survival in IDH wt glioma and the mesenchymal subtype of GBM.

**Figure S5 related to Figure 2.** Functional analysis in IDH wt glioma revealing a close correlation between SASP Score elevation and the activation of indicated signaling pathways associated with tumorigenesis.

**Figure S6 related to Figure 3.** Functional analysis revealing a close correlation between SASP Score elevation and dysregulated immune responses in IDH wt glioma.

**Figure S7 related to Figure 3.** GSEA analysis of cancer hallmarks revealing a significant association between SASP Score elevation and dysregulated immune response in IDH wt glioma.

**Figure S8 related to Figure 3.** SASP Score elevation indicates dysregulation of immune response in IDH wt glioma.

**Figure S9 related to Figure 3.** SASP Score elevation implicates extensive infiltration of macrophages in the immunosuppressive tumor microenvironment of IDH wt glioma.

**Figure S10 related to Figure 5.** Small molecular inhibitor screening identifies GDC-0879 as a potential SASP inhibitor in glioma.

**Figure S11 related to Figure 5.** GDC-0879 serves as a potential SASP inhibitor effectively reducing GBM cell proliferation in vitro.

**Figure S12 related to Figure 6.** Doxorubicin significantly induces senescence and SASP activation in THP1-derived macrophages and mouse BMDMs.

**Figure S13 related to Figure 7.** Increased SASP Score indicates a poor response to immunotherapy in cancer.

**Figure S14 related to Figure 7.** Elevated SASP Score are more predominantly enriched in the single cell samples from non-responder to anti-PD1 therapy in GBM (GSE235676).

**Figure S15 related to Figure 7.** GDC-0879 treatment significantly reduces GBM cell tumorigenicity and improves their responses to PD1 blockade in preclinical GL261 orthotopic mice model.

**Figure S16 related to Figure 7.** GDC-0879 treatment increases apoptosis ratios of GBM cells and macrophages in mice orthotopic xenograft models.

**Figure S17 related to Figure 7.** GDC-0879 sensitizes GBM to anti-PD1 immunotherapy in preclinical GL261 orthotopic mice model.

**Figure S18 related to Figure 7.** The in vivo administration of GDC-0879 doesn’t lead to significant liver and kidney damage in mice orthotopic xenograft model.

**Figure S19 related to Figure 7.** Representative immunohistochemical staining and analysis of indicated MAPK signaling pathway components in intracranial xenograft sections from indicated tumor bearing mice groups.

**Figure S20 related to Figure 7.** Elevated SASP Score are enriched in GBM samples with tumor treating field (TTF) and control treatment.

**Supplementary Figures and Figure legends**


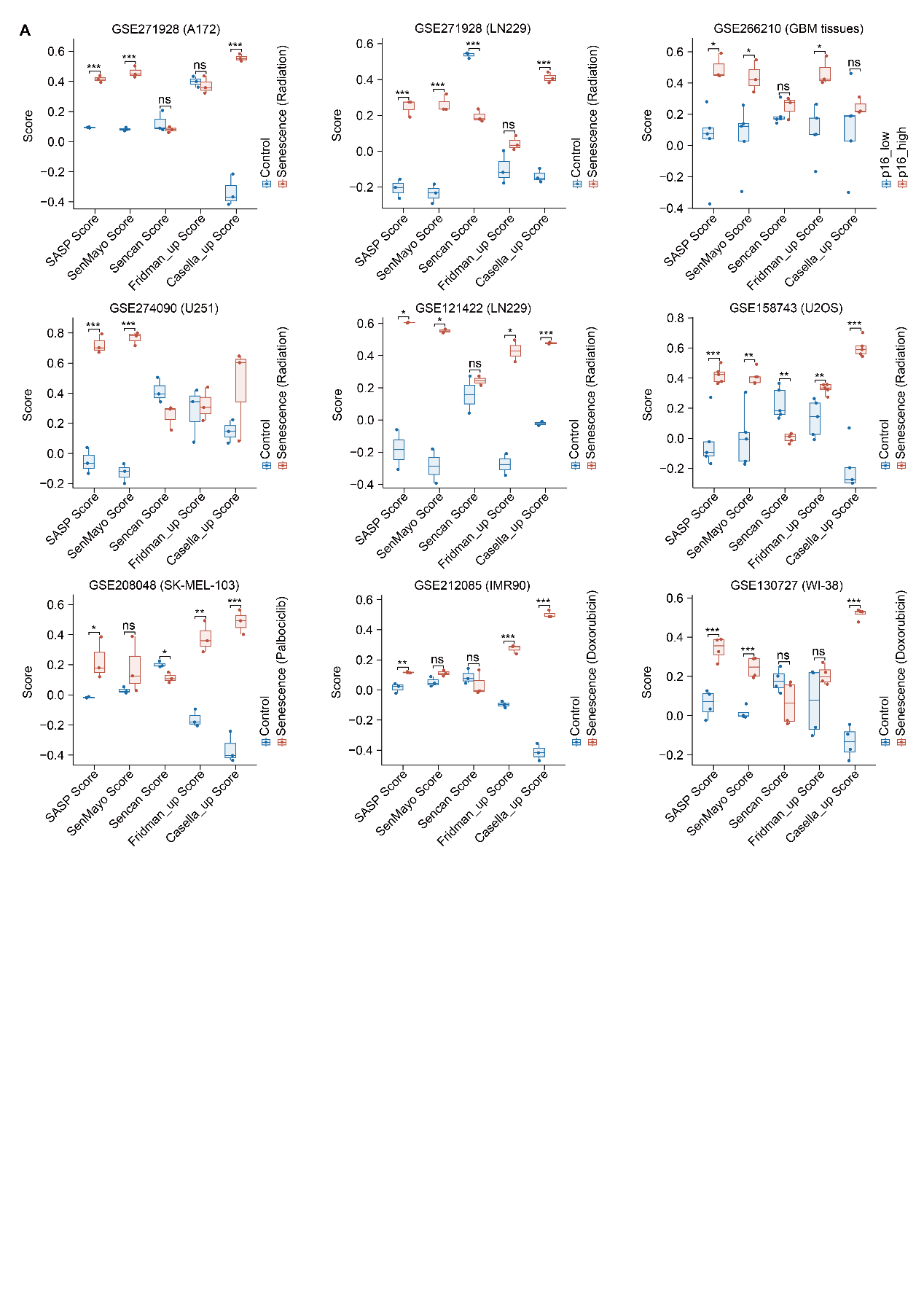
  **Figure S1 related to Figure 1. Comparison of current SASP gene panel with four published senescence gene sets in senescence evaluation according to indicated datasets.**

**A.** The comparison of five senescence score established by current SASP gene panel and other four reported senescence gene sets with ssGSEA method, respectively, in eight senescence GEO datasets (GSE271928: A172 n = 6 and LN229 n = 6, radiation-induced senescence in A172 and LN229 GBM cells; GSE266210: n = 8, p16_high (senescence) and p16_low (non-senescence) in GBM tissues; GSE274090: n = 6, radiation-induced senescence in U251 GBM cells; GSE121422: n = 4, radiation-induced senescence in LN229 GBM cells; GSE158743: n = 10, radiation-induced senescence in U2OS human osteosarcoma cells; GSE208048: n = 6, palbociclib-induced senescence in SK-MEL-103 melanoma cells; GSE212085: n = 6, doxorubicin-induced senescence in IMR90 fibroblast cells; GSE130727: n = 8, doxorubicin-induced senescence in WI-38 fibroblast cells).

(ns not significant, * P < .05, ** P < .01, *** P < .001)


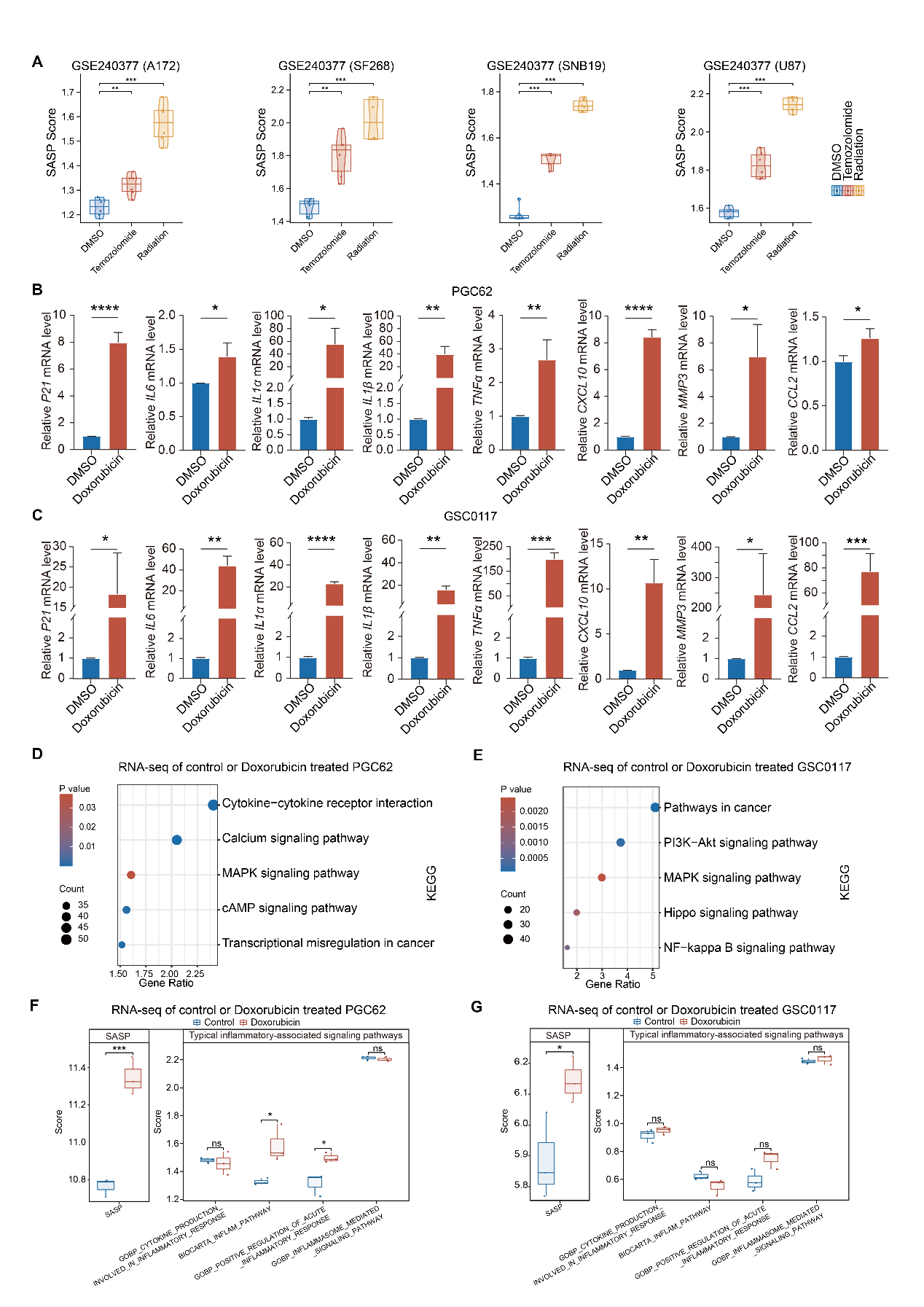


**Figure S2 related to Figure 1. Elevated SASP Score efficiently represents SASP activation in GBM cells.**

**A.** SASP scoring of indicated four senescence induced GBM cell lines (GSE240377, n = 69, senescence induced by TMZ or radiotherapy, one-way ANOVA test).

**B, C.** RT-qPCR analysis of indicated SASP-related genes in control and Doxorubicin-induced senescence GBM cell samples (A: PGC62 and B: GSC0117) (n = 3, t-test).

**D, E.** Kyoto Encyclopedia of Genes and Genomes Pathway-based (KEGG) enrichment analysis of RNA-seq data obtained from indicated control and Doxorubicin-induced senescence GBM cell samples (C: PGC62 and D: GSC0117).

**F, G.** The comparative analysis of current SASP Score and four typical inflammatory state scores establishing by corresponding gene sets and ssGSEA method, according to RNA-seq data obtained from PGC62 (E) and GSC0117 (F) samples with control or Doxorubicin (200 nM) treatment (n = 3).

(* P < .05, ** P < .01, *** P < .001, **** P < .0001, ns not significant.)


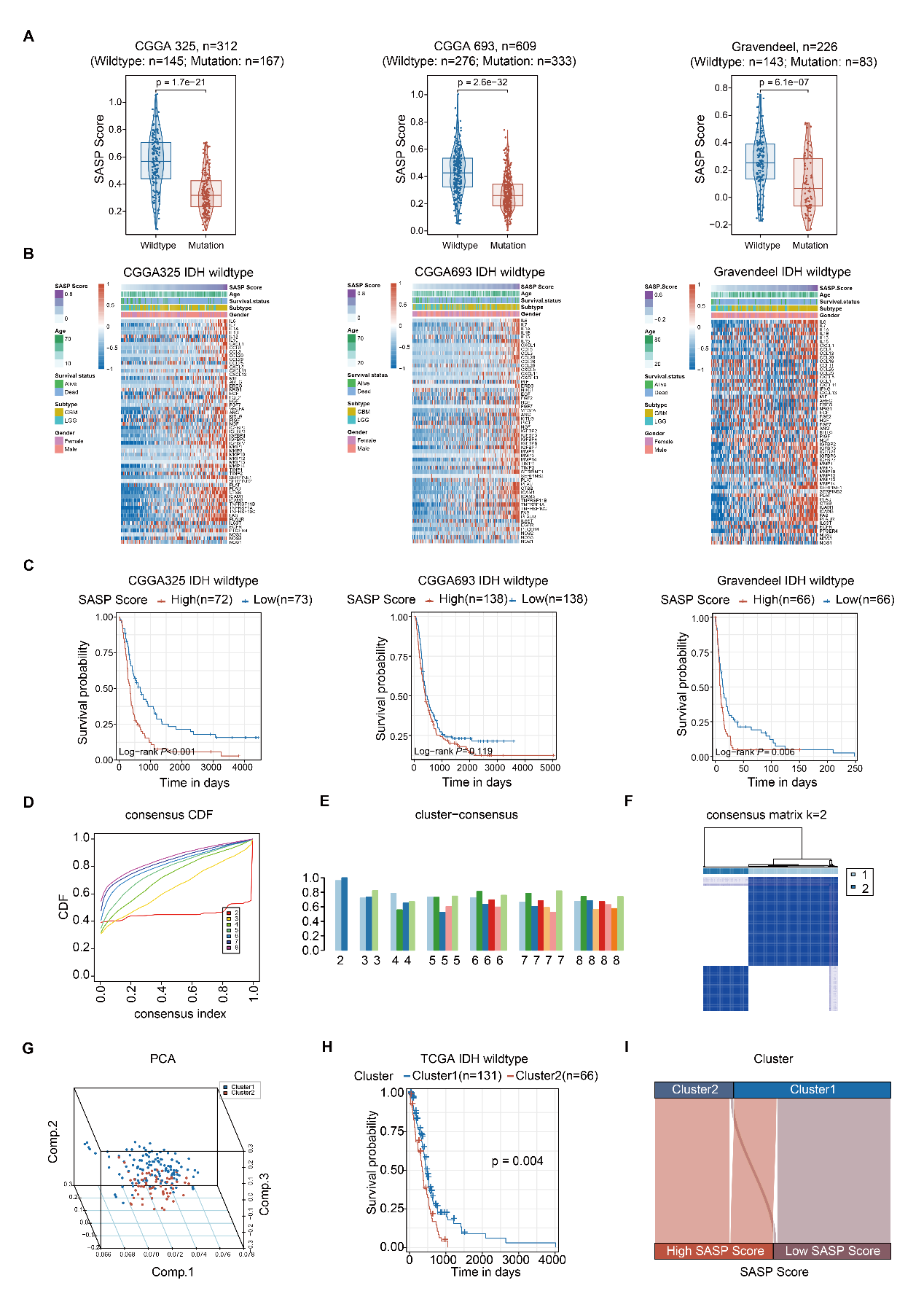


**Figure S3 related to Figure 2. SASP activation is intimately associated with the malignant progression of IDH wt glioma.**

**A.** The analysis based on established SASP Score showing that IDH wt glioma exhibited a higher SASP Score than IDH mut glioma (CGGA325 glioma: IDH wild type n = 145, Mutation n = 167; CGGA693 glioma: IDH wild type n = 276, Mutation n = 333, Gravendeel glioma: IDH wild type n = 143, Mutation n = 83).

**B.** The analysis of indicated glioma datasets showing that SASP Score elevation reflected the upregulation of indicated SASP associated genes. (CGGA325, IDH wt glioma n = 145; CGGA693, IDH wt glioma n = 276; Gravendeel, IDH wt glioma n = 136, 7 cases with not applicable grade information weren’t included in the analysis).

**C.** Kaplan-Meier analyses revealing that SASP Score elevation was correlated with unfavorable prognosis in indicated IDH wt glioma cohorts (CGGA325, IDH wt glioma n = 145: high n = 72, low n = 73; CGGA693, IDH wt glioma n = 276: high n = 138, low n = 138; Gravendeel, IDH wt glioma n = 132: high n = 66, low n = 66, 7 cases with not applicable grade information and 4 cases with not applicable survival information weren’t included in the analysis; Log-rank test).

**D-F.** The clustering analysis based on the status of SASP activation in IDH wt glioma (TCGA, n = 197). Consensus clustering CDF for k = 2 to 8 (D); The consensus score matrix of all samples when k= 2 (E); Cluster-consensus plot for k = 2 to 8 (F).

**G.** Principal component analysis (PCA) based on SASP Score revealing that SASP activation effectively identified the malignant progression of IDH wt glioma (TCGA, IDH wt glioma n = 197).

**H.** Survival analysis demonstrating favorable survival in Cluster 1 with lower SASP Score, in comparison with Cluster 2 with higher SASP Score (TCGA, IDH wt glioma n = 197: Cluster 1 n = 131, Cluster 2 n = 66).

**I.** Sankey diagram depicting the relationship between indicated Clusters and SASP Score (TCGA, IDH wt glioma n = 197: Cluster 1 n = 125, Cluster 2 n = 72).


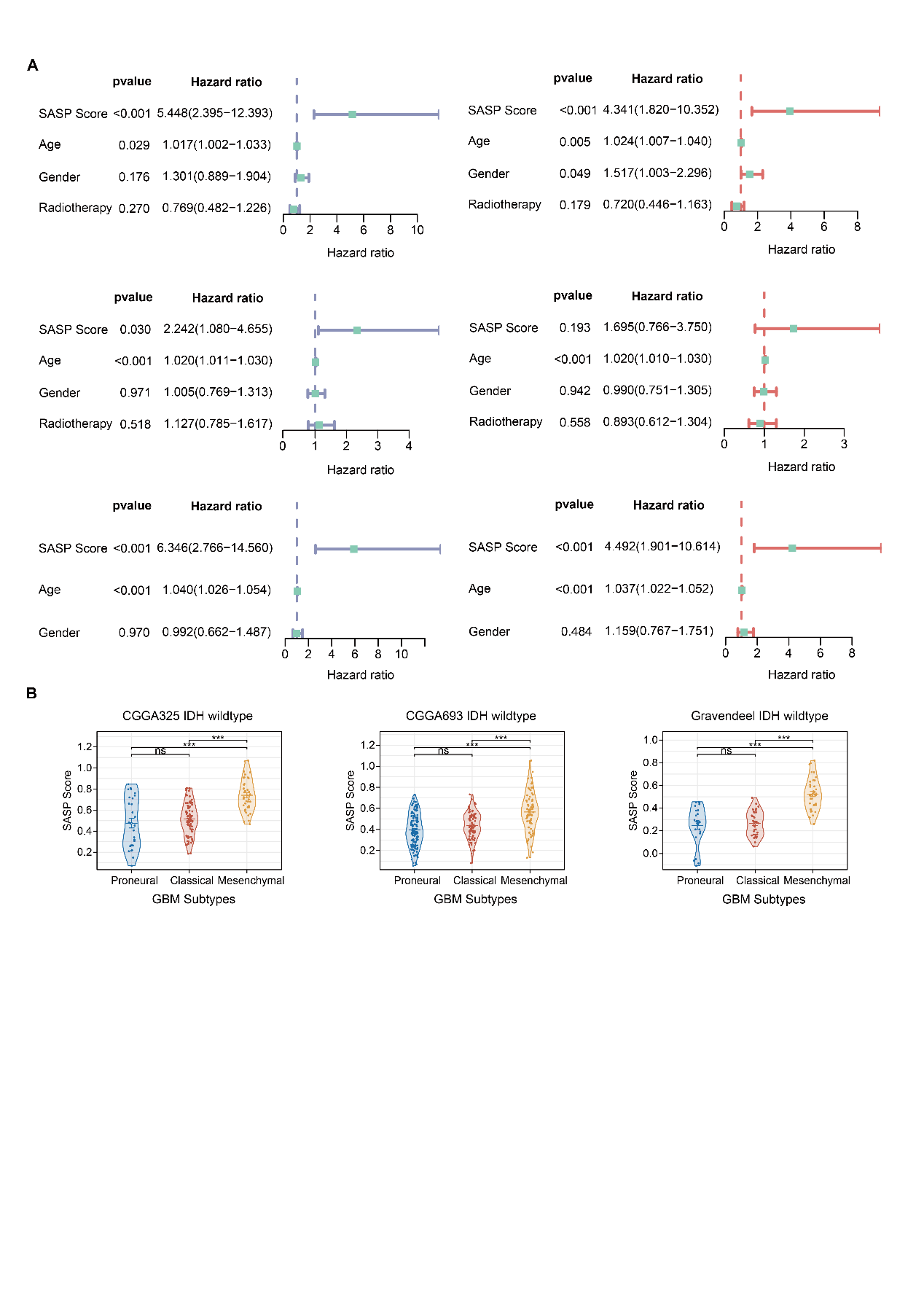


**Figure S4 related to Figure 2. SASP Score elevation is closely correlated with poor survival in IDH wt glioma and the mesenchymal subtype of GBM.**

**A.** The univariate (Left) and multivariate (Right) analyses indicating that SASP Score elevation was an independent prognosis factor of IDH wt glioma (Upper: CGGA325, IDH wt glioma n = 145; middle: CGGA693, IDH wt glioma n = 276; lower: Gravendeel, IDH wt glioma, n = 132, 7 cases with not applicable grade information and 4 cases with not applicable survival information weren’t included in the analysis).

**B.** The SASP Score evaluation in proneural, classical, and mesenchymal subtypes of GBM (Left: CGGA325, GBM n = 145: proneural n = 28, classical n = 78, and mesenchymal n = 39; middle: CGGA693, GBM n = 276: proneural n = 122, classical n = 81, and mesenchymal n = 73; lower: Gravendeel, GBM n = 95: proneural n = 25, classical n = 34, and mesenchymal n = 36; Wilcoxon rank sum test).

(* P < .05, ** P < .01, *** P < .001, **** P < .0001, ns not significant.)


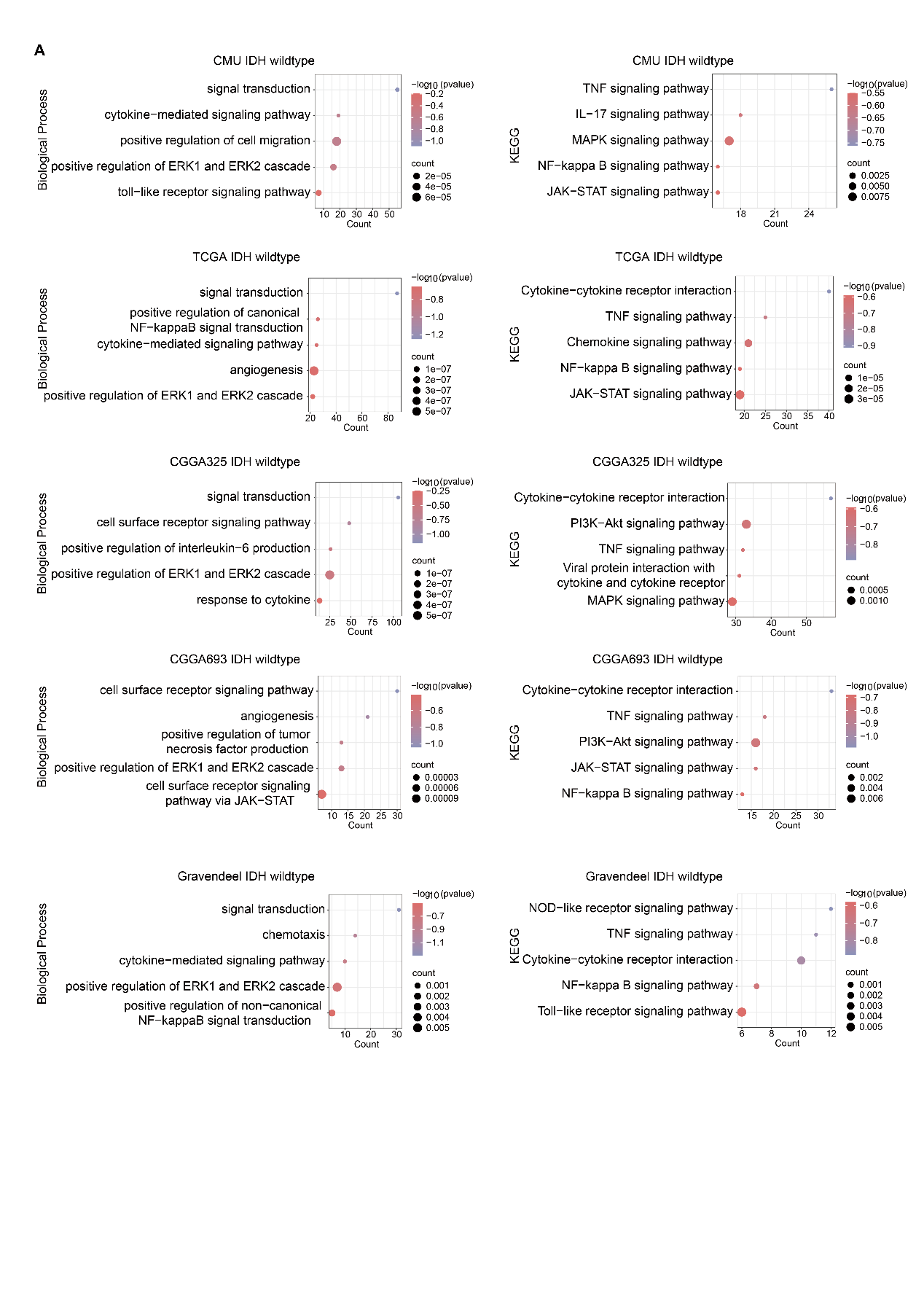


**Figure S5 related to Figure 2. Functional analysis in IDH wt glioma revealing a close correlation between SASP Score elevation and the activation of indicated signaling pathways associated with tumorigenesis.**

**A.** GO biological process (Left) and KEGG Pathway (Right) analysis in IDH wt glioma disclosing the enrichment of indicated biological processes associated with cancer initiation and progression in high SASP Score samples (DAVID; TCGA, IDH wt glioma n = 197; CGGA325, IDH wt glioma n = 145; CGGA693, IDH wt glioma n = 276; Gravendeel, IDH wt glioma n = 136, 7 cases with not applicable grade information weren’t included in the analysis; R > 0.6, Spearman correlation analysis).


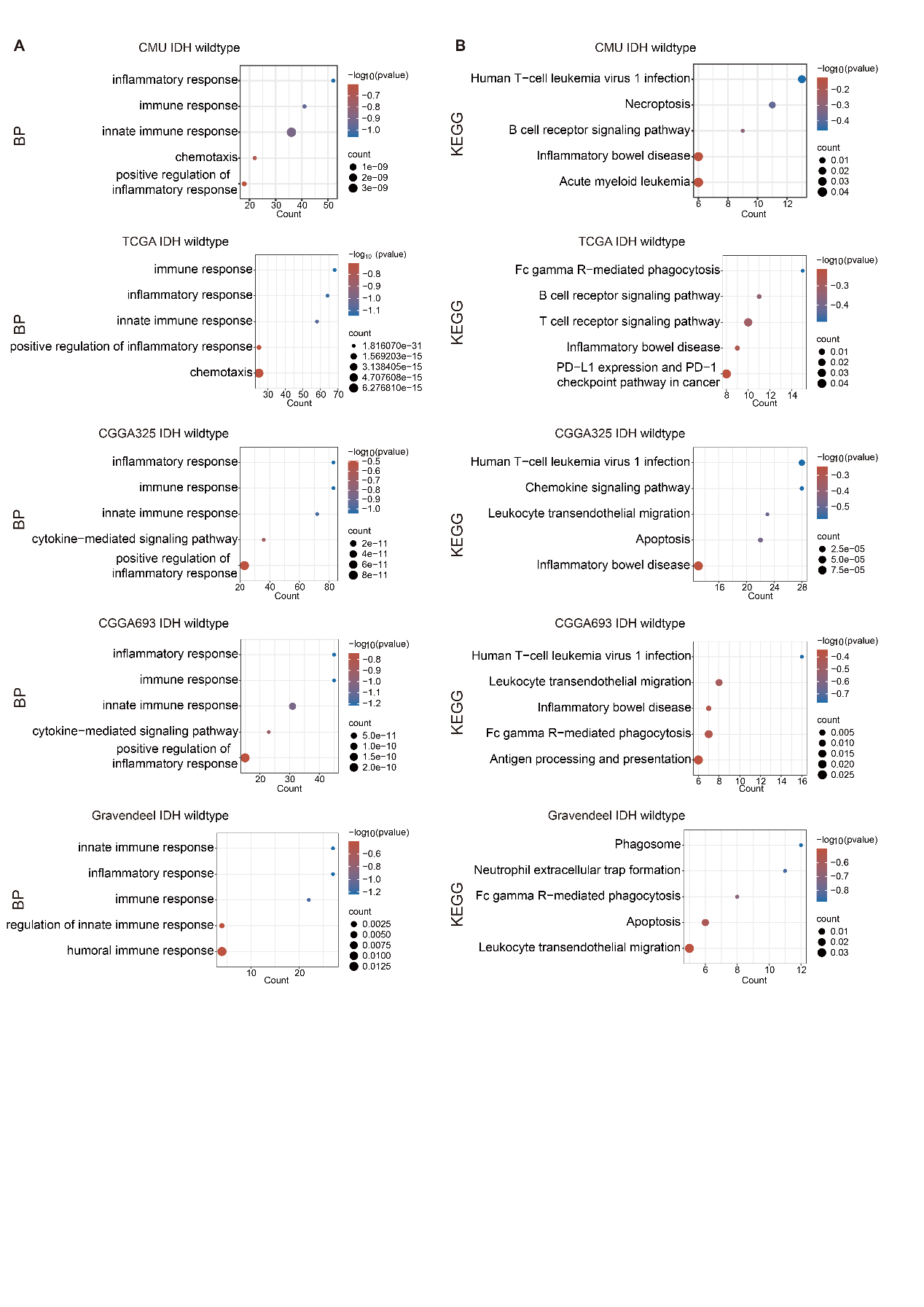


**Figure S6 related to Figure 3. Functional analysis revealing a close correlation between SASP Score elevation and dysregulated immune responses in IDH wt glioma.**

**A, B.** GO biological process (A) and KEGG pathway (B) analysis in IDH wt glioma samples (TCGA, IDH wt glioma n = 197; CGGA325, IDH wt glioma n = 145; CGGA693, IDH wt glioma n = 276; Gravendeel, IDH wt glioma n = 136, 7 cases with not applicable grade information weren’t included in the analysis; R > 0.6, Spearman correlation analysis)


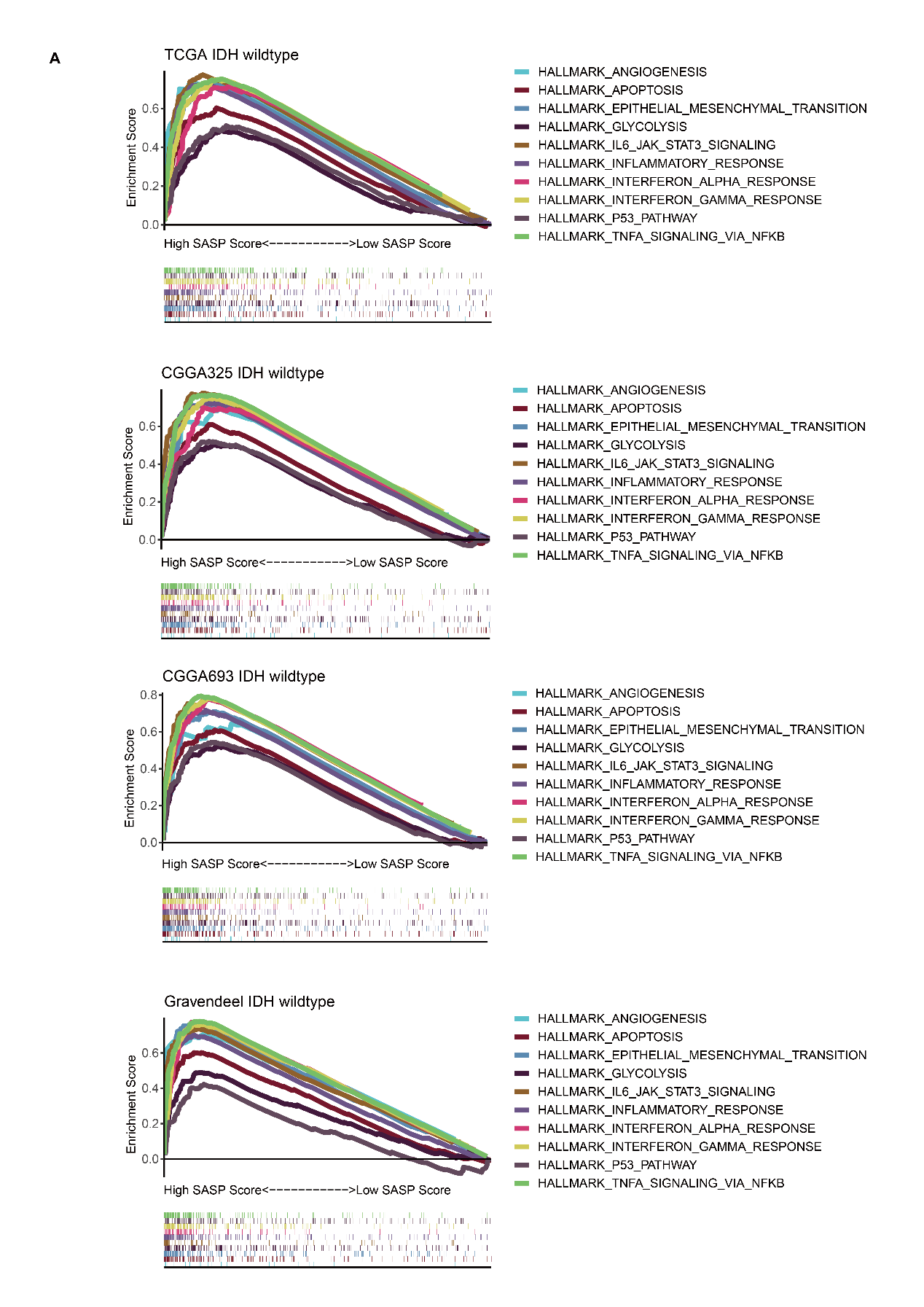


**Figure S7 related to Figure 3. GSEA analysis of cancer hallmarks revealing a significant association between SASP Score elevation and dysregulated immune response in IDH wt glioma.**

**A.** GSEA analysis of indicated cancer hallmarks in IDH wt Glioma (TCGA, IDH wt glioma n = 197: high n = 99, low n = 98; CGGA325, IDH wt glioma n = 145: high n = 72, low n = 73;; CGGA693, IDH wt glioma n = 276: high n = 138, low n = 138; Gravendeel, IDH wt glioma n = 136: high n = 68, low n = 68, 7 cases with not applicable grade information weren’t included in the analysis, NES and FDR q-val are supplemented in Table S9).


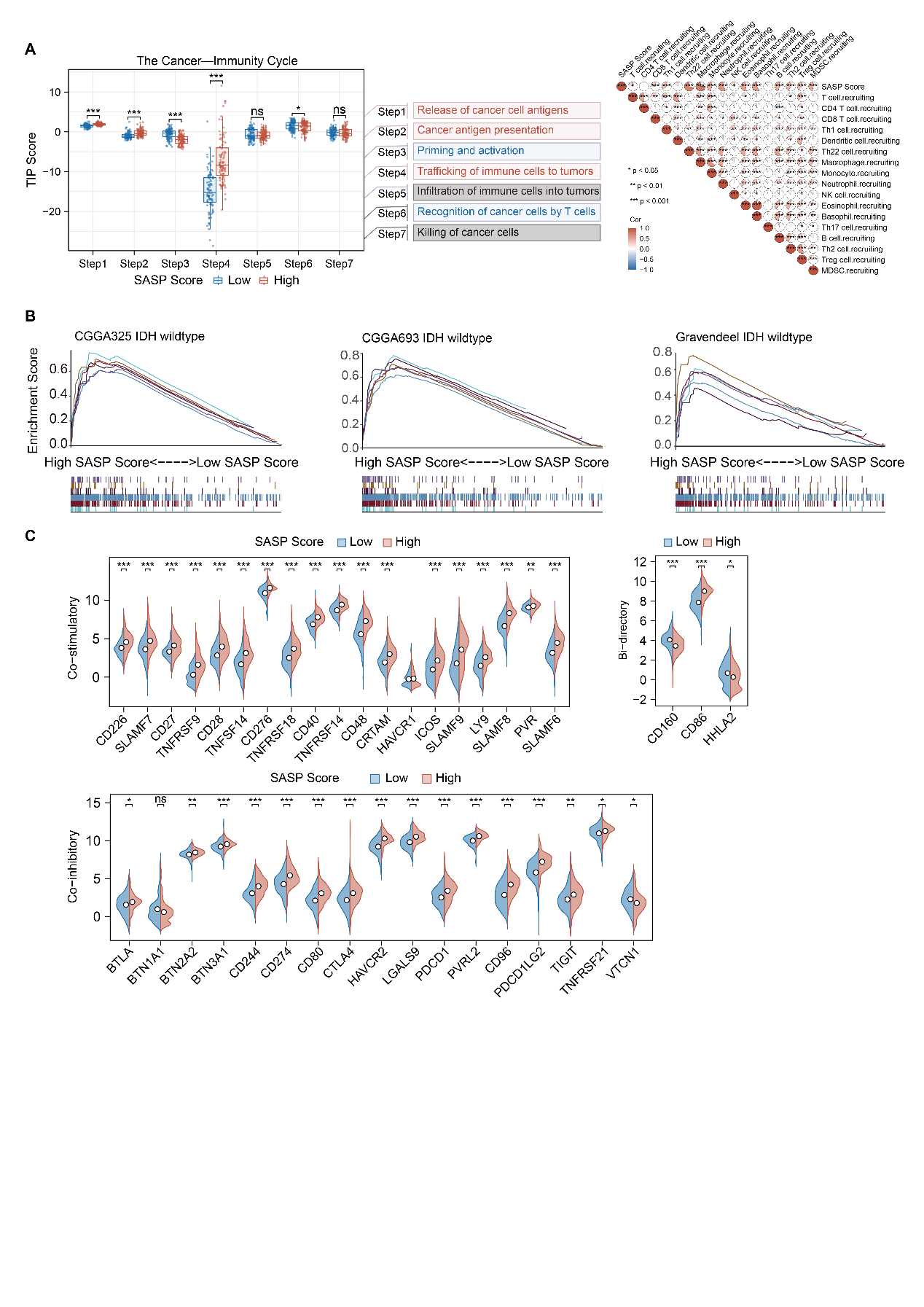


**Figure S8 related to Figure 3. SASP Score elevation indicates dysregulation of immune response in IDH wt glioma.**

**A.** The evaluation of Tracking Tumor Immunophenotype (TIP) score in IDH wt glioma samples with high and low SASP Score (TCGA IDH wt glioma, n = 197: high n = 99, low n = 98; t-test), according to seven steps of the Cancer-Immunity Cycle (TIP: http://biocc.hrbmu.edu.cn/TIP/).

**B.** GSEA analysis revealing a potential stronger negative regulation of immune responses in IDH wt glioma samples with high SASP Score (CGGA325 IDH wt glioma, n = 145: high n = 73, low n = 72; CGGA693, IDH wt glioma n = 276: high n = 138, low n = 138; Gravendeel, IDH wt glioma n = 136: high n = 68, low n = 68, 7 cases with not applicable grade information weren’t included in the analysis).

**C.** The expression analysis of indicated immune checkpoints in IDH wt glioma samples stratified with SASP Score (TCGA IDH wt glioma n = 197: high n = 99, low n = 98; t-test).

(ns not significant, * P < .05, ** P < .01, *** P < .001)


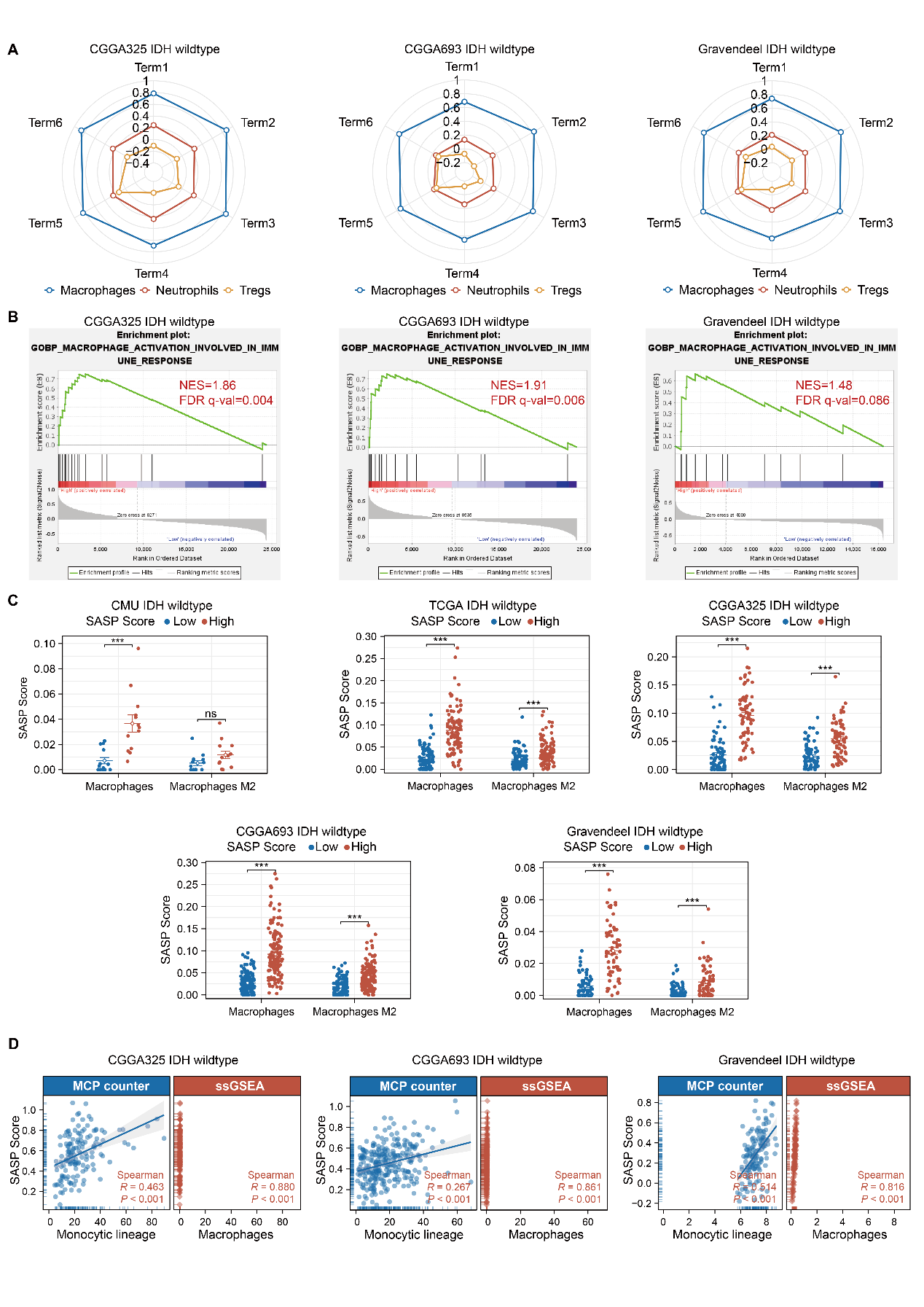


**Figure S9 related to Figure 3. SASP Score elevation implicates extensive infiltration of macrophages in the immunosuppressive tumor microenvironment of IDH wt glioma.**

**A.** The correlation analysis of indicated non-malignant immunosuppressive cell populations and indicated immunosuppressive terms in IDH wt glioma (CGGA325, IDH wt glioma n = 145; CGGA693, IDH wt glioma n = 276; Gravendeel, IDH wt glioma n = 136, 7 cases with not applicable grade information weren’t included in the analysis; Spearman correlation analysis).

**B.** GSEA analysis in IDH wt glioma revealing a significant correlation of SASP Score elevation to macrophage activation involved in immune responses (CGGA325 IDH wt glioma, n = 145: high n = 73, low n = 72; CGGA693, IDH wt glioma n = 276: high n = 138, low n = 138; Gravendeel, IDH wt glioma n = 136: high n = 68, low n = 68, 7 cases with not applicable grade information weren’t included in the analysis).

**C.** The analysis based on Xcell method indicating enhanced infiltration of tumor associated macrophages (TAMs), especially M2 TAMs, in high SASP Score IDH wt glioma samples compared to low SASP Score specimens (CMU, IDH wt Glioma n = 26; TCGA, IDH wt Glioma n = 197; CGGA325, IDH wt Glioma n = 145; CGGA693, IDH wt Glioma n = 276; Gravendeel, IDH wt Glioma n = 136, 7 cases with not applicable grade information weren’t included in the analysis).

**D.** The correlation analysis of SASP Score and monocytic lineage based on MCP counter method, as well as that of SASP score and macrophages based on ssGSEA method (CGGA325, IDH wt Glioma n = 145; CGGA693, IDH wt Glioma n = 276; Gravendeel, IDH wt Glioma n = 136, 7 cases with not applicable grade information weren’t included in the analysis).

(ns not significant, *** P < .001)


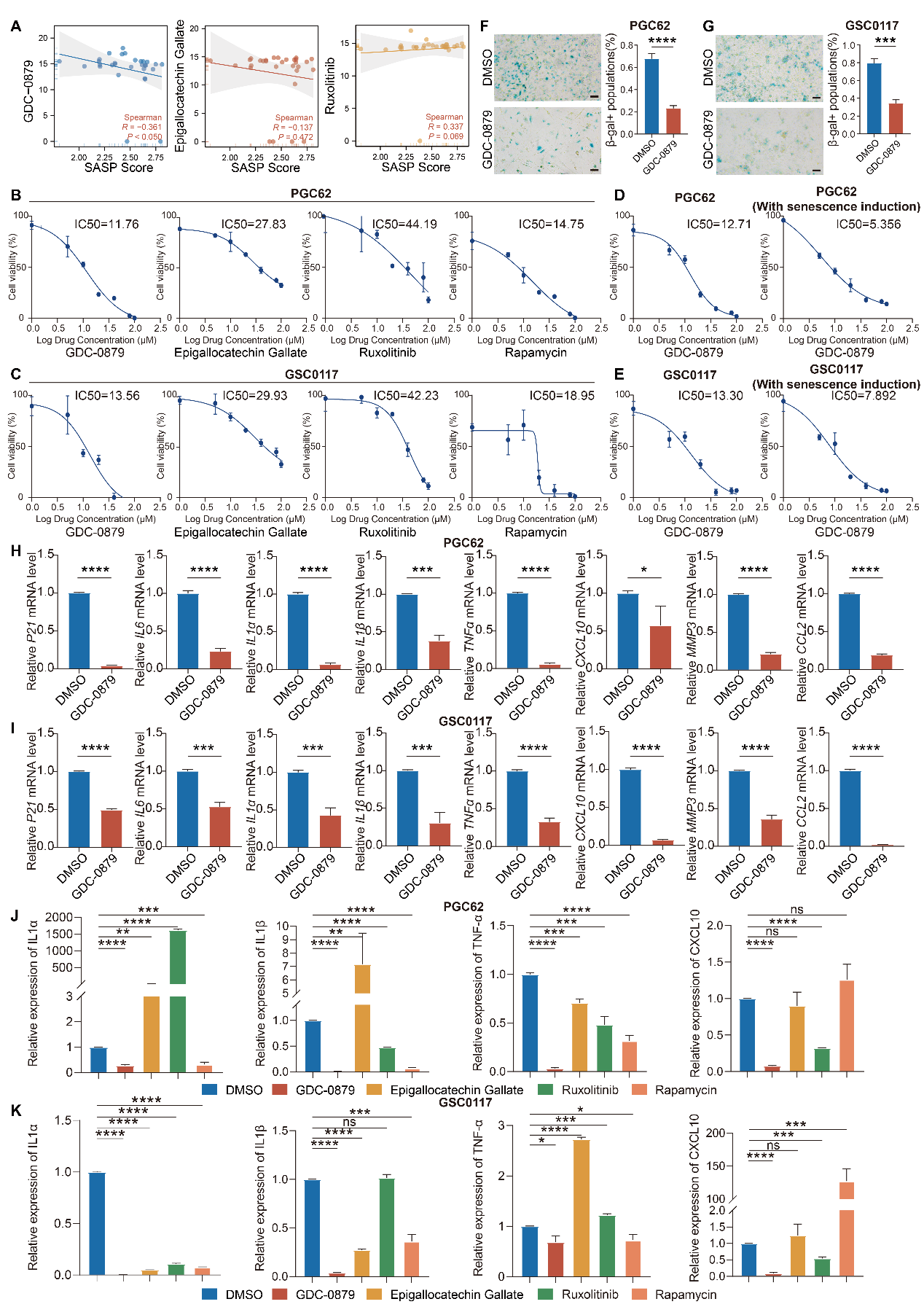


**Figure S10 related to Figure 5. Small molecular inhibitor screening identifies GDC-0879 as a potential SASP inhibitor in glioma.**

**A.** Scatter plots showing the correlation between SASP Score and AUC values of indicated drugs in 30 glioma cell lines from Cancer Cell Line Encyclopedia (CCLE). Spearman correlation coefficients and P values are shown.

**B, C.** IC50 analysis of PGC62 (B) and GSC0117 (C) GBM cells to indicated SASP inhibitors (n = 3).

**D, E.** IC50 analysis of indicated GBM cells (PGC62 (D) and GSC0117 (E)) and senescence GBM cells (doxorubicin induced senescence) to GDC-0879 (n = 3).

**F, G.** Representative SA-β-gal staining images (Left) and analysis (Right) of GDC-0879 treated senescence GBM cells (F: PGC62 and G: GSC0117; GDC-0879 10 μM) (n = 3, t-test). Scale bar, 50 μm.

**H, I.** RT-qPCR analysis of indicated SASP marker mRNA in control (DMSO) or senescence PGC62 (H) and GSC0117 (I) samples induced by Doxorubicin and then with or without GDC-0879 treatment (10 μM) (P21, IL-6, IL-1α, IL1β, TNF-α, CXCL10, MMP3, and CCL2; n = 3, t-test).

**J, K.** RT-qPCR analysis of indicated SASP marker mRNA in senescence PGC62 (J) and GSC0117 (K) samples induced by Doxorubicin and then treated with GDC-0879, Epigallocatechin Gallate, Ruxolitinib, and Rapamycin at their IC50 concentration, respectively (Left: IL1α; Left middle: IL1β; Right middle: TNF-α; Right: CXCL10; n = 3, t test).

(ns not significant, * P < .05, ** P < .01, *** P < .001, **** P < .0001)
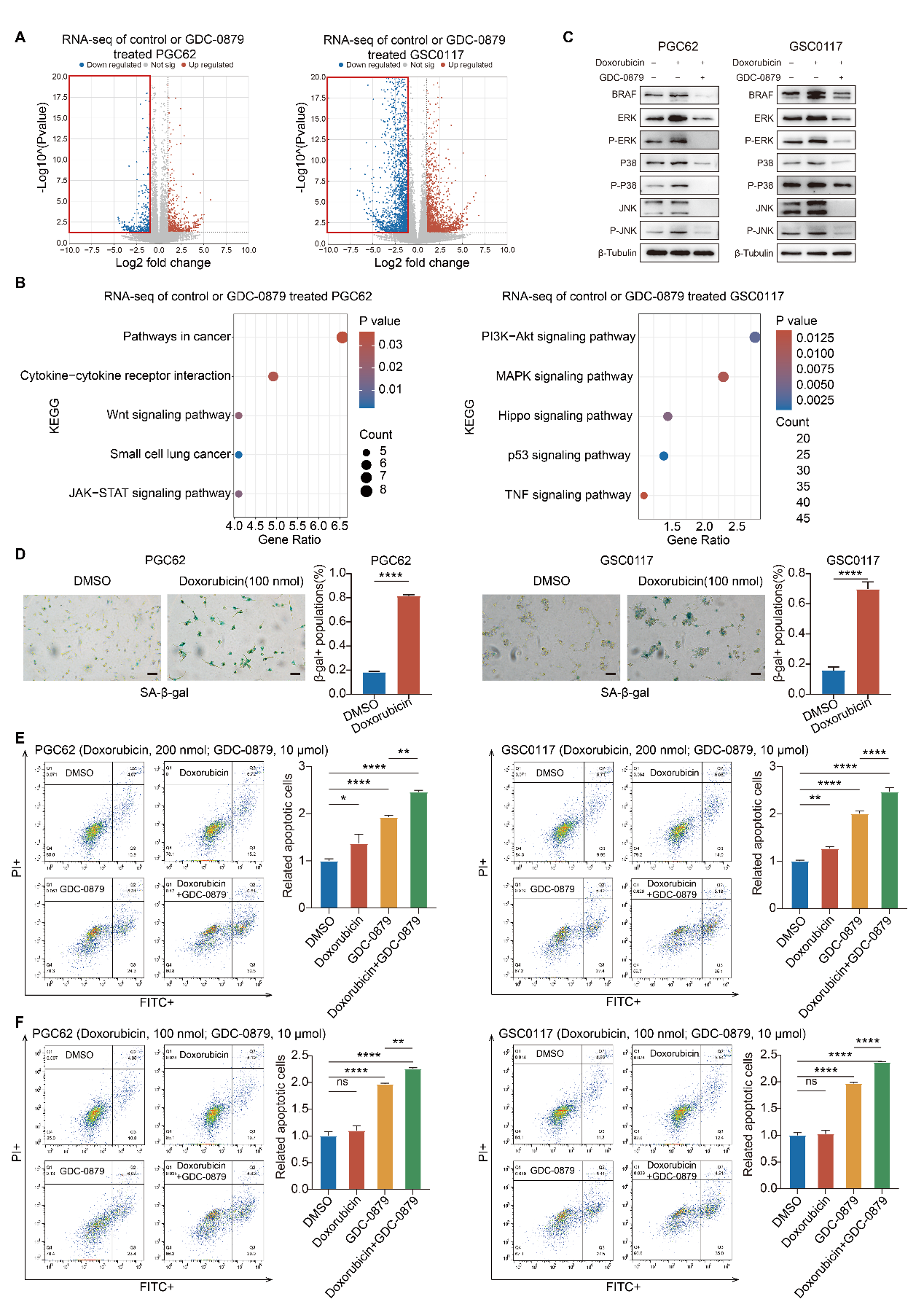


**Figure S11 related to Figure 5. GDC-0879 serves as a potential SASP inhibitor effectively reducing GBM cell proliferation in vitro.**

**A.** The analysis of differential expressed genes (DEGs) with RNA-seq data obtained from indicated GBM cell samples treated with or without GDC-0879 (Left: PGC62; right: GSC0117; with DESeq2).

**B.** KEGG analysis of RNA-seq data obtained from control and Doxorubicin treated PGC62 (Upper) and GSC0117 samples (Lower).

**C.** Immunoblotting analyses of indicated MAPK signaling targets in indicated control and doxorubicin (200 nM for 48 h to induce senescence, and senescence cell samples were collected at 5 days after senescence induction) treated PGC62 (Left) and GSC0117 (Right) samples followed with or without GDC-0879 treatment (10 µM treatment for 48 h)

**D.** Representative SA-β-gal staining images (Left) and analysis (Right) of GBM cells with DMSO or 100 nM Doxorubicin treatment (n = 3, t-test). Scale bar, 50 μm.

**E, F** Flow-cytometry analysis of apoptotic cell ratio in indicated GBM cell samples with or without GDC-0879 treatment (DMSO: PGC62 or GSC0117 treated with DMSO; Doxorubicin: PGC62 or GSC0117 treated with 200 nM (E) or 100 nM (F) Doxorubicin; GDC-0879: PGC62 or GSC0117 treated with 10 μM GDC-0879; Doxorubicin+GDC-0879: 200 nM (E) or 100 nM (F) Doxorubicin pretreated PGC62 or GSC0117, which were treated with 10 μM GDC-0879 after Doxorubicin pretreatment to induce senescence; n = 3, one-way ANOVA).

(ns not significant, * P < .05, ** P < .01, *** P < .001, **** P < .0001)


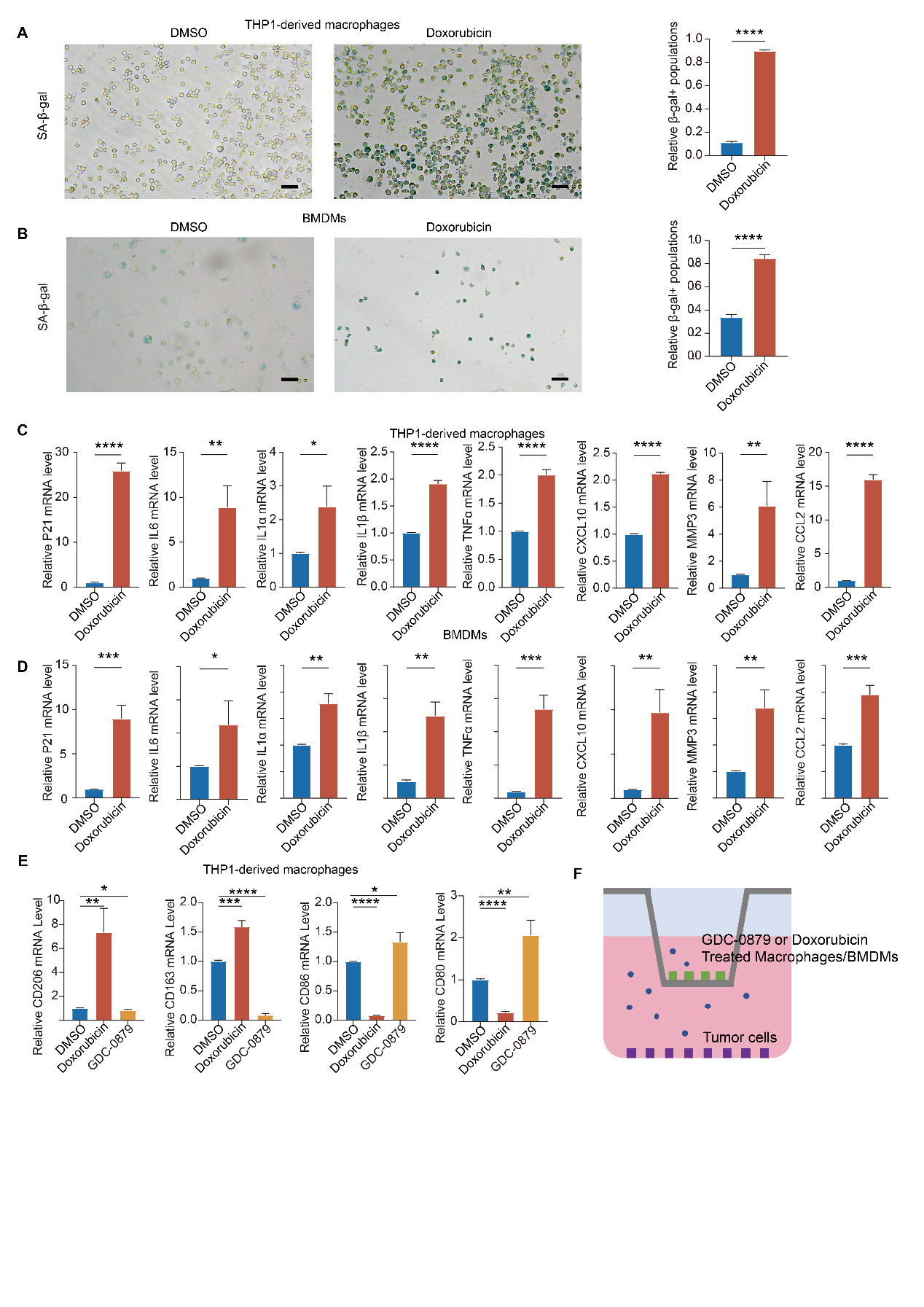


**Figure S12 related to Figure 6.** **Doxorubicin significantly induces senescence and SASP activation in THP1-derived macrophages and mouse BMDMs.**

**A, B.** Representative SA-β-gal staining images (Left) and analysis (Right) of THP-1 derived macrophages (A) and mouse BMDMs (B) treated with or without indicated Doxorubicin treatment (200 nM, n = 3, t-test). Scale bar, 50 μm.

**C, D.** RT-qPCR analysis of indicated SASP-related marker genes in THP-1 derived macrophages (C) and mouse BMDMs (D) treated with DMSO or Doxorubicin (200 nM), respectively (P21, IL-6, IL-1α, IL1β, TNF-α, CXCL10, MMP3, and CCL2; n = 3, t-test).

**E.** RT-qPCR analysis of indicated TAM markers (CD206, CD163, CD86, and CD80) in THP-1 derived macrophages with indicated treatment (DMSO, 200 nM Doxorubicin, or 10 μM GDC-0879; n = 3, t-test).

**F.** The schematic graph showing the co-culture method of untreated glioma cells with the coculture of control or Doxorubicin (200 nM) pretreated THP-1/BMDMs derived TAMs with or without GDC-0879 treatment, respectively.

(ns not significant, * P < .05, ** P < .01, *** P < .001, **** P < .0001)


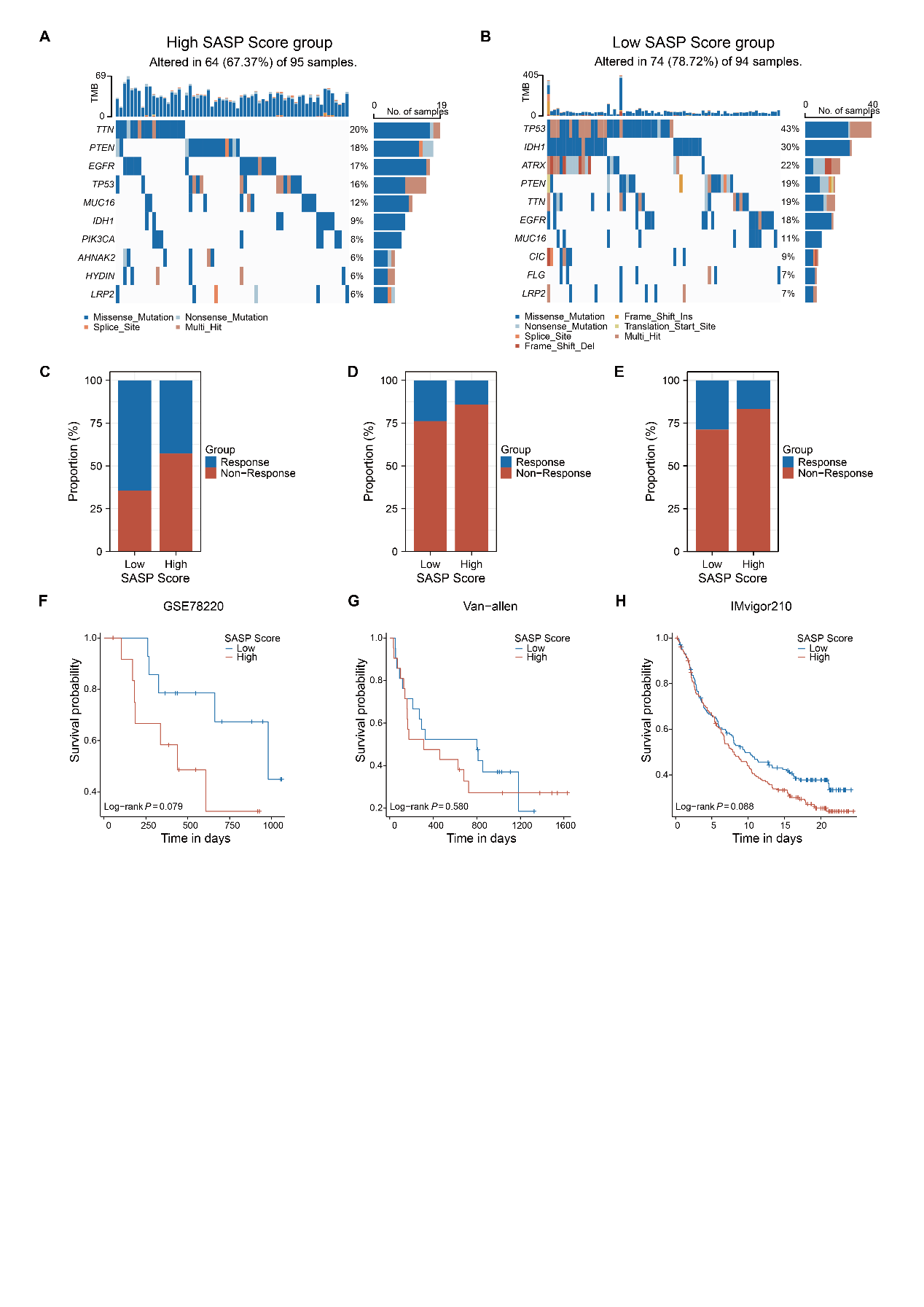


**Figure S13 related to Figure 7. Increased SASP Score indicates a poor response to immunotherapy in cancer.**

**A, B.** Somatic mutation analysis in TCGA IDH wt glioma samples stratified with SASP Score (A. High SASP Score group n = 95; B. Low SASP Score group n = 94; Mutation information NA samples weren’t included in the analysis).

**C-E.** SASP Score and therapeutic response analyses in Melanoma datasets (C. GSE78220, with anti-PD1: High SASP Score group n = 14, Low SASP Score group n = 14; D. Van-allen, with anti-CTL4: High SASP Score group n = 21, Low SASP Score group n = 21) and IMvigor210 metastatic urothelial cancer dataset (E. with anti-PDL1: High SASP Score group n = 149, Low SASP Score group n = 149, 50 cases with not applicable therapeutic response information weren’t included in the analysis).

**F-H.** The survival analysis in indicated cohorts stratified with SASP Score (F. GSE78220, with anti-PD1, High SASP Score group n = 13, Low SASP Score group n = 14, 1 cases with not applicable survival information weren’t included in the analysis; G. Van-Allen, with anti-CTL4, High SASP Score group n =21, Low SASP Score group n = 21; H. IMvigor210 dataset, with anti-PDL1, High SASP Score group n = 174, Low SASP Score group n = 174, Log-rank test).


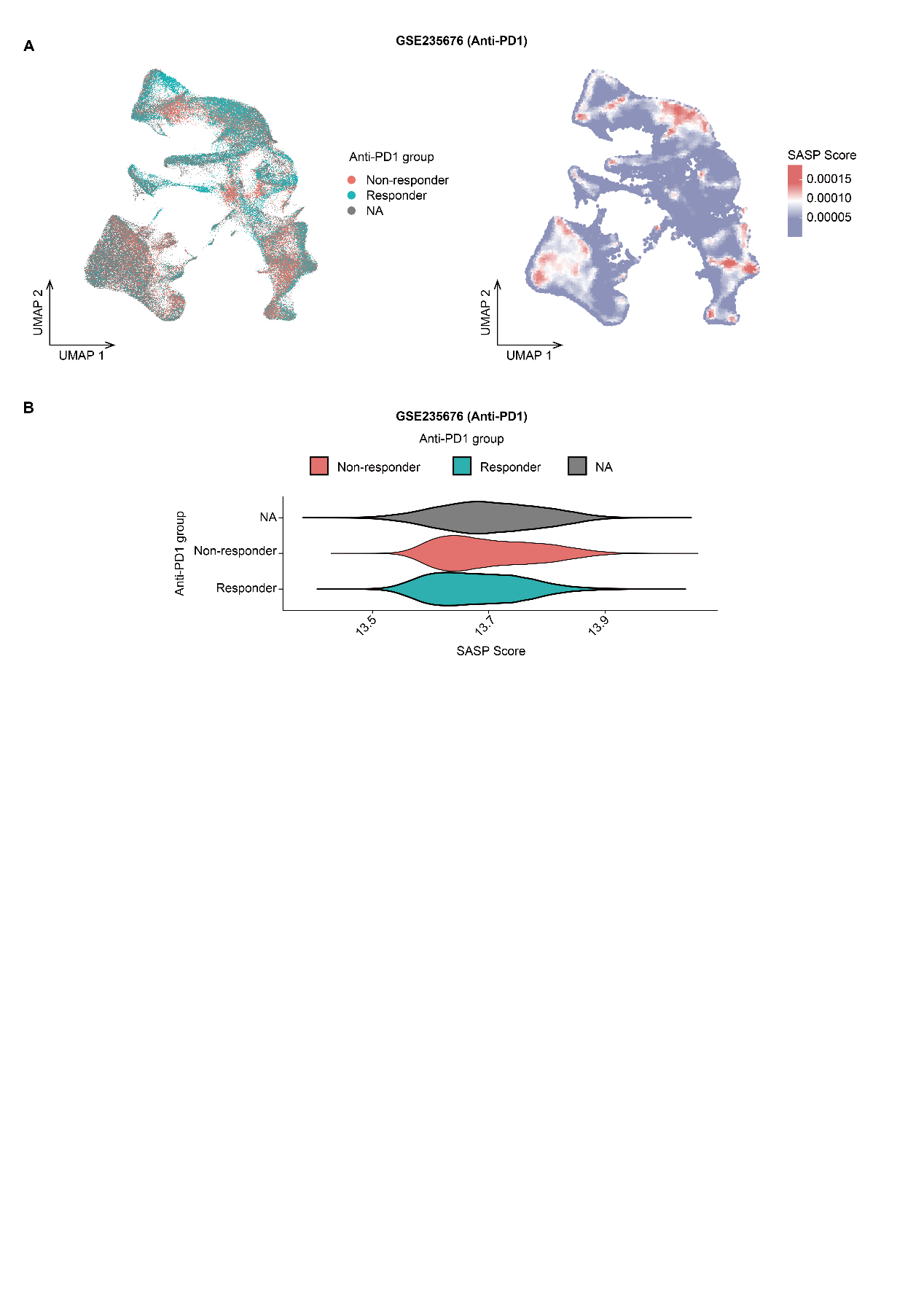


**Figure S14 related to Figure 7. Elevated SASP Score are more predominantly enriched in the single cell samples from non-responder to anti-PD1 therapy in GBM (GSE235676).**

**A.** The UMAP plot of 149,048 cells (Left) from 24 tumor samples analyzed by single cell RNA-seq (GSE235676) and corresponding SASP Score profile (Right) (GBM patients were stratified by the responding to anti-PD1 therapy).

**B.** The SASP Score analysis of single cell samples from GBM patients stratified by the responding to anti-PD1 therapy according to GSE235676 GBM single cell RNA-seq dataset (Responder, n = 56039; Non-responder, n = 48652; NA, n = 44357).


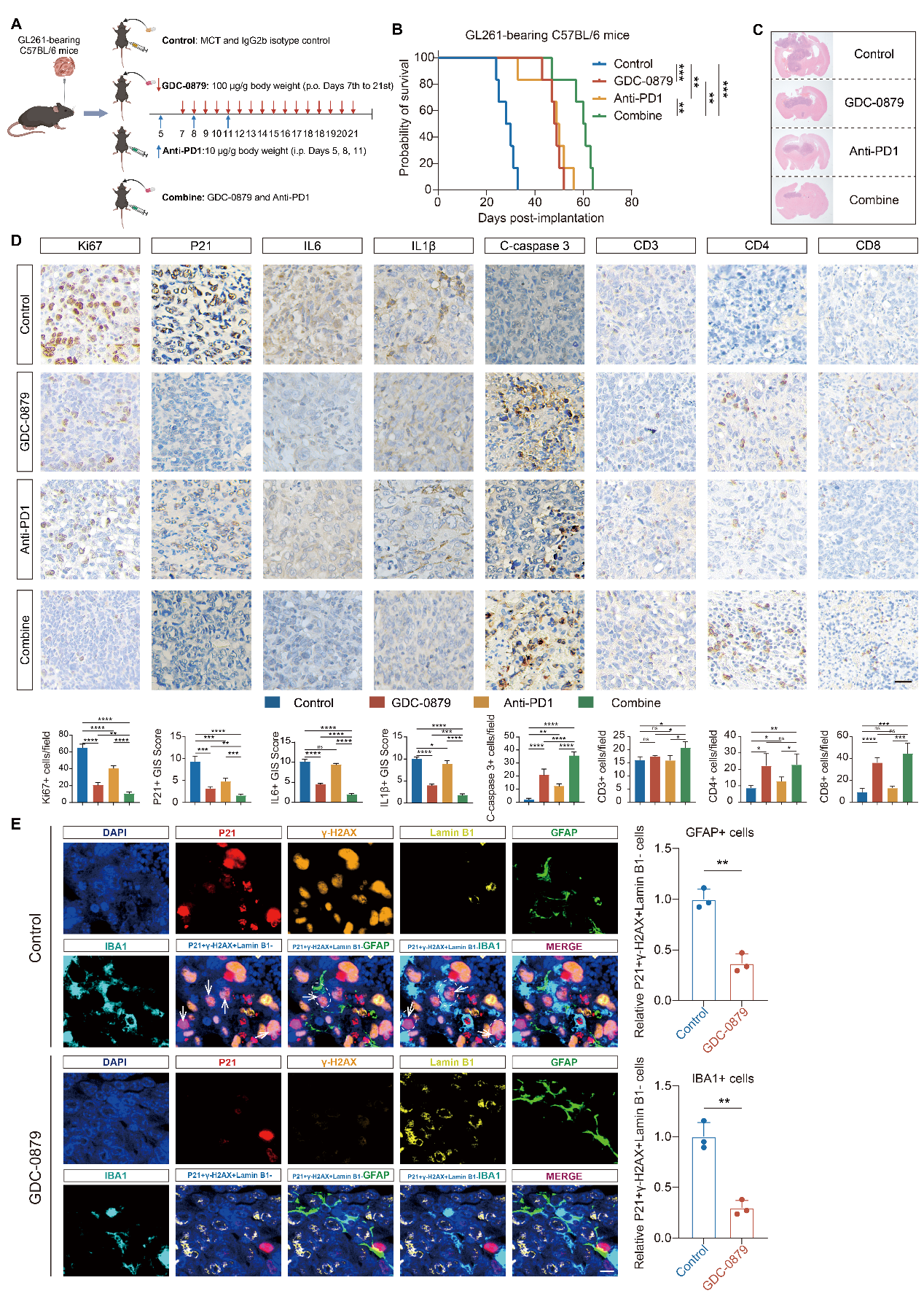


**Figure S15 related to Figure 7. GDC-0879 treatment significantly reduces GBM cell tumorigenicity and improves their responses to PD1 blockade in preclinical GL261 orthotopic mice model.**

**A.** The schematic graph showing the administration method of indicated treatment in GL261 mouse GBM orthotopic model. Figure S15A was Created in BioRender.

**B.** Survival plot of mice intracranially transplanted with GL261 cells and then receiving indicated treatment (Control: receiving a single oral dose of 0.5% methylcellulose/0.2% Tween 80 (MCT) and IgG2b isotype control; GDC-0879: GDC-0879 (100 μg/g body weight) in MCT was orally administered by gavage daily from 7th to 21st day after intracranial implantation of GL-261 cells; Anti-PD1: Anti-PD1 antibody (10 μg/g body weight) was injected intraperitoneally on the 5th, 8th, and 11th day after intracranial implantation of GL-261 cells; Combined treatment: treated with GDC-0879 and anti-PD1; Log-rank test, n = 6).

**C.** Representative H&E staining images of brain sections from indicated mice groups on day 21 after intracranial transplantation of GL261 cells.

**D.** Representative immunohistochemical staining images and analysis of P21, IL6, IL1β, C-caspase-3 (cleaved Caspase-3), CD3, CD4, and CD8 in GL261 derived intracranial xenograft sections from indicated mice groups (n = 4, one-way ANOVA). Scale bar, 25 μm.

**E.** Representative immunofluorescence images of DAPI (Dark blue), P21 (Red), γ-H2AX (Orange), Lamin B1 (Yellow), GFAP (Green), and IBA1 (Cyan) in GL261 derived intracranial xenograft sections from indicated mice groups (n = 3, t test). Scale bar, 10 μm.

(ns not significant, * P < .05, ** P < .01, *** P < .001, **** P < .0001)


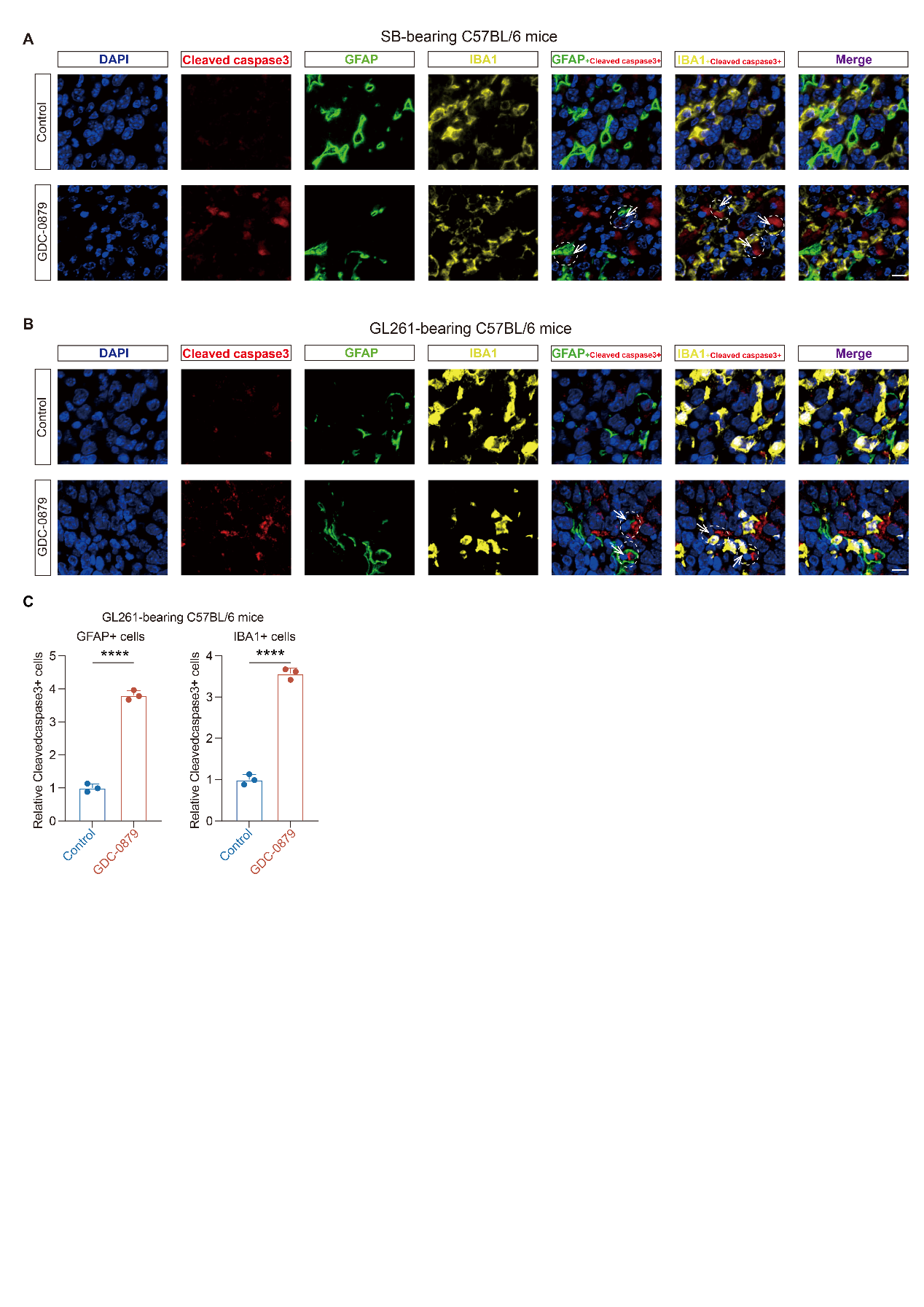


**Figure S16 related to Figure 7. GDC-0879 treatment increases apoptosis ratios of GBM cells and macrophages in mice orthotopic xenograft models.**

**A, B.** Representative immunofluorescence images of DAPI (Dark blue), Cleaved caspase3 (Red), GFAP (Green), and IBA1 (Yellow) in SB mouse GBM cell (A, related to Figure 7F) and GL261 (B) derived intracranial xenograft from indicated mice groups (Related to Figure 7F). Scale bar, 10 μm.

**C.** The analysis of cell with positive expression of GFAP and cleaved caspase 3, and IBA1 and cleaved caspase 3, respectively, according to immunofluorescence staining of GL261 derived intracranial xenograft sections from indicated mice groups (n = 3, t test).


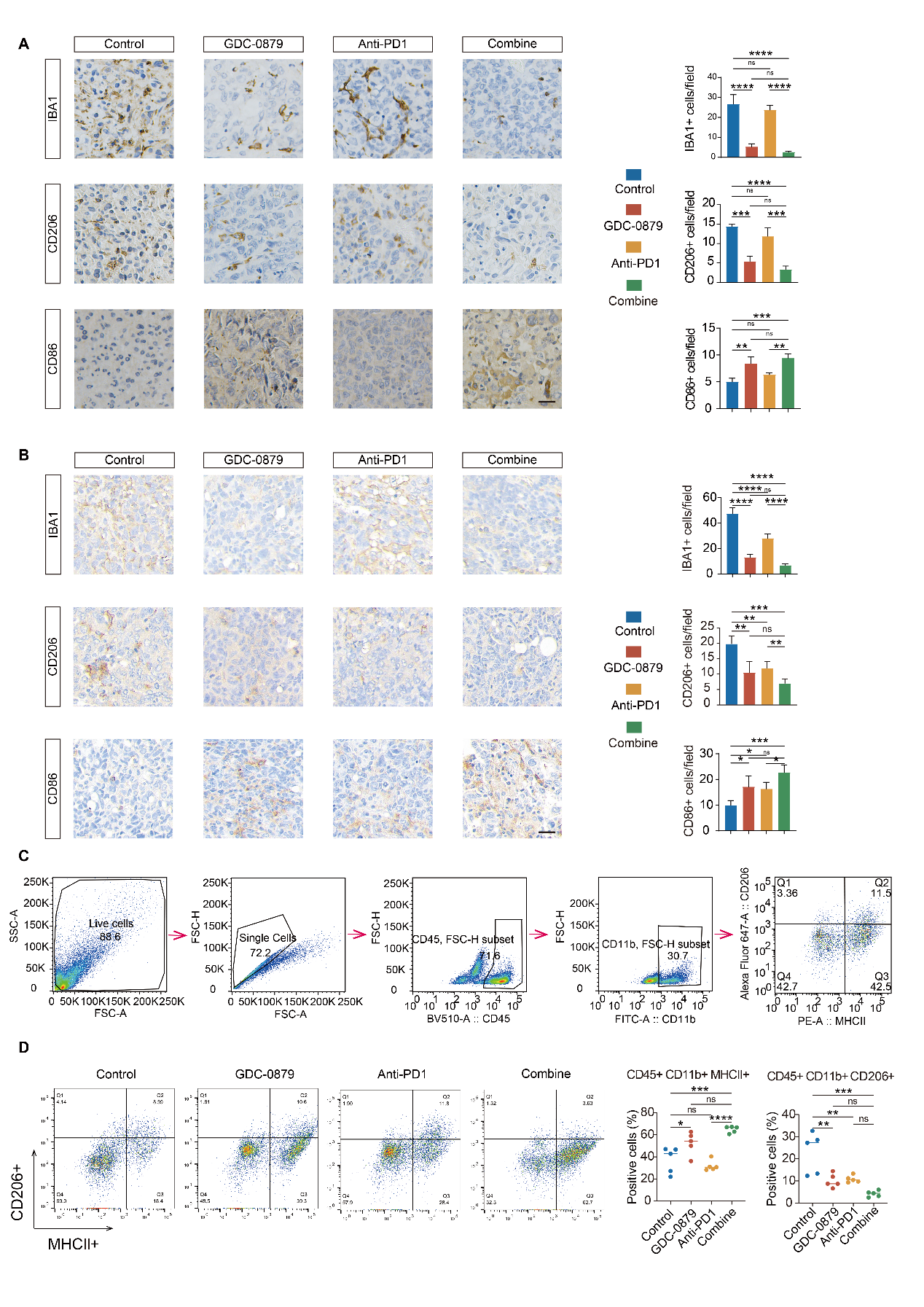


**Figure S17 related to Figure 7. GDC-0879 sensitizes GBM to anti-PD1 immunotherapy in preclinical GL261 orthotopic mice model.**

**A, B.** Representative immunohistochemical staining images (Left) and analyses (Right) of IBA1, CD206, and CD86 (SB mouse GBM cell (A, n = 3) and GL261 (B, n = 4) derived intracranial xenograft. Upper: IBA1, middle: CD206, and lower: CD86) in intracranial xenograft sections from indicated mice groups (one-way ANOVA). Scale bar, 25 μm.

**C.** Flow-cytometry gating strategies for quantifying CD206^+^ and MHC-II^+^ cells in tumor samples from GL261-bearing C57BL6 mice (Related to Figure S17D).

**D.** FACS analysis of indicated markers (CD206 and MHC-II) in GL261 intracranial xenograft samples from indicated mice groups (n = 5, one-way ANOVA).

(ns not significant, * P < .05, ** P < .01, *** P < .001, **** P < .0001)


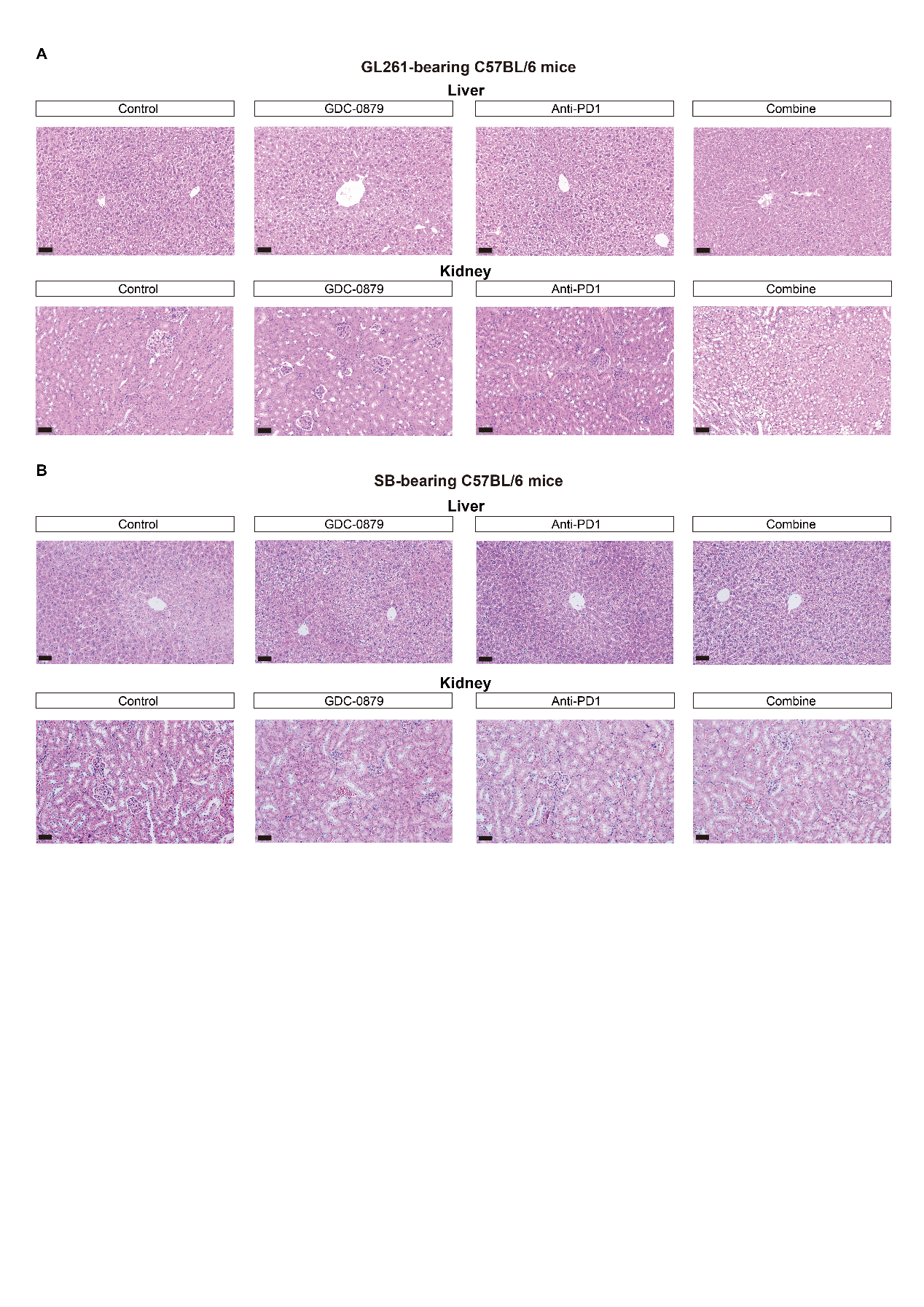


**Figure S18 related to Figure 7. The in vivo administration of GDC-0879 doesn’t lead to significant liver and kidney damage in mice orthotopic xenograft model.**

**A, B.** Representative H&E staining images of liver (Upper) and kidney (Lower) sections from GL261 (A) or SB mouse GBM cell (B) derived orthotopic xenograft bearing C57BL/6 mice receiving control, GDC-0879, anti-PD1, or combined treatment. Scale bar, 50 μm. (Control: receiving a single oral dose of 0.5% methylcellulose/0.2% Tween 80 (MCT) and IgG2b isotype control; GDC-0879: GDC-0879 (100 μg/g body weight) in MCT was orally administered by gavage daily from 7th to 21st day after intracranial implantation of GBM cells; Anti-PD1: Anti-PD1 antibody (10 μg/g body weight) was injected intraperitoneally on the 5th, 8th, and 11th day after intracranial implantation of GBM cells; Combined treatment: treated with GDC-0879 and anti-PD1).


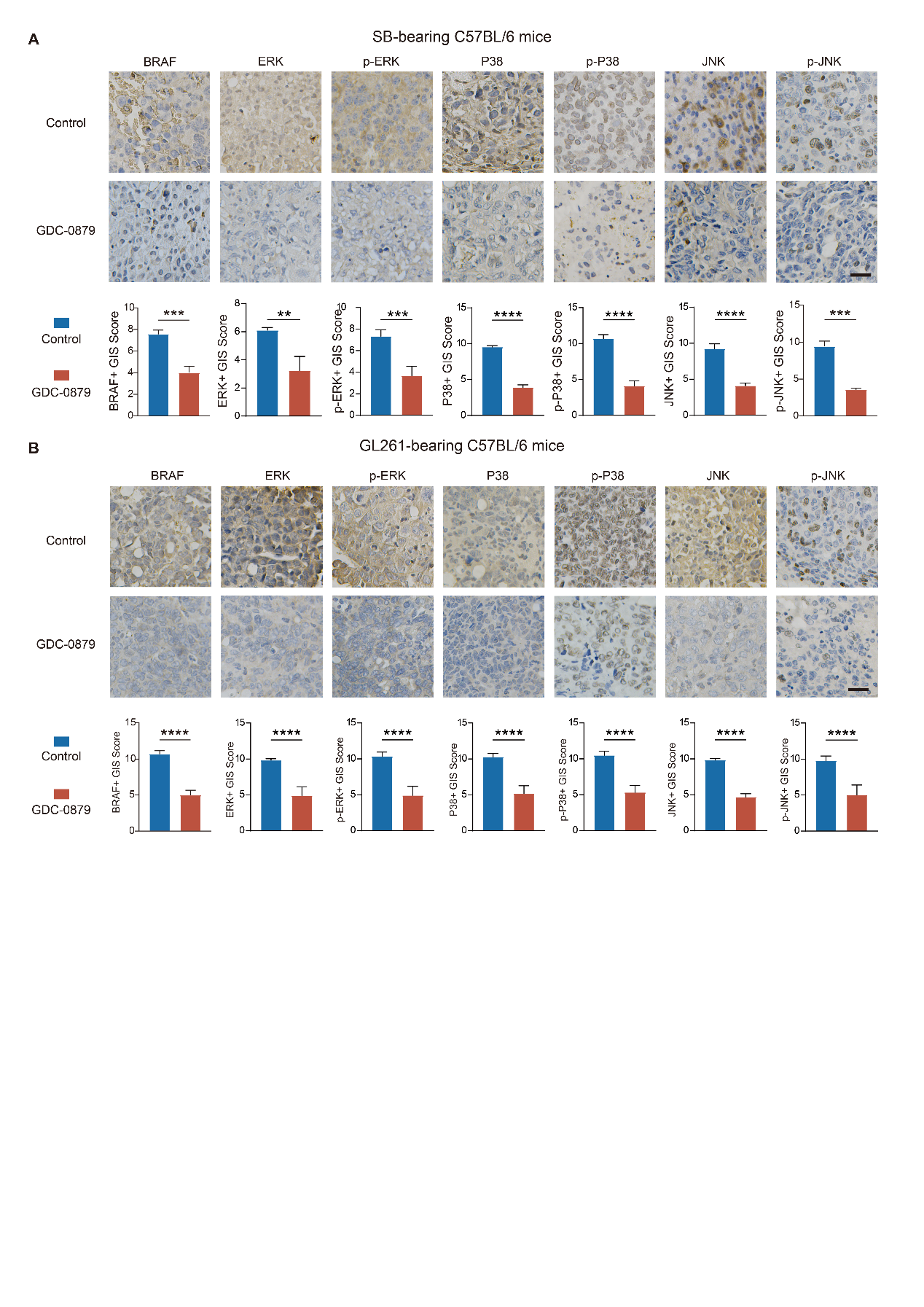


**Figure S19 related to Figure 7. Representative immunohistochemical staining and analysis of indicated MAPK signaling pathway components in intracranial xenograft sections from indicated tumor bearing mice groups.**

**A, B.** Representative immunohistochemical staining (Upper and middle) and analysis (Lower) of MAPK signaling pathway components, including BRAF, ERK, p-ERK, P38, p-P38, JNK and p-JNK, in SB mouse GBM cell (A) and GL261 (B) derived intracranial xenograft sections from indicated tumor bearing mice groups (Upper, control; middle, GDC-0879; n = 3, t test). Scale bar, 25 μm.

(ns not significant, ** P < .01, *** P < .001, **** P < .0001)


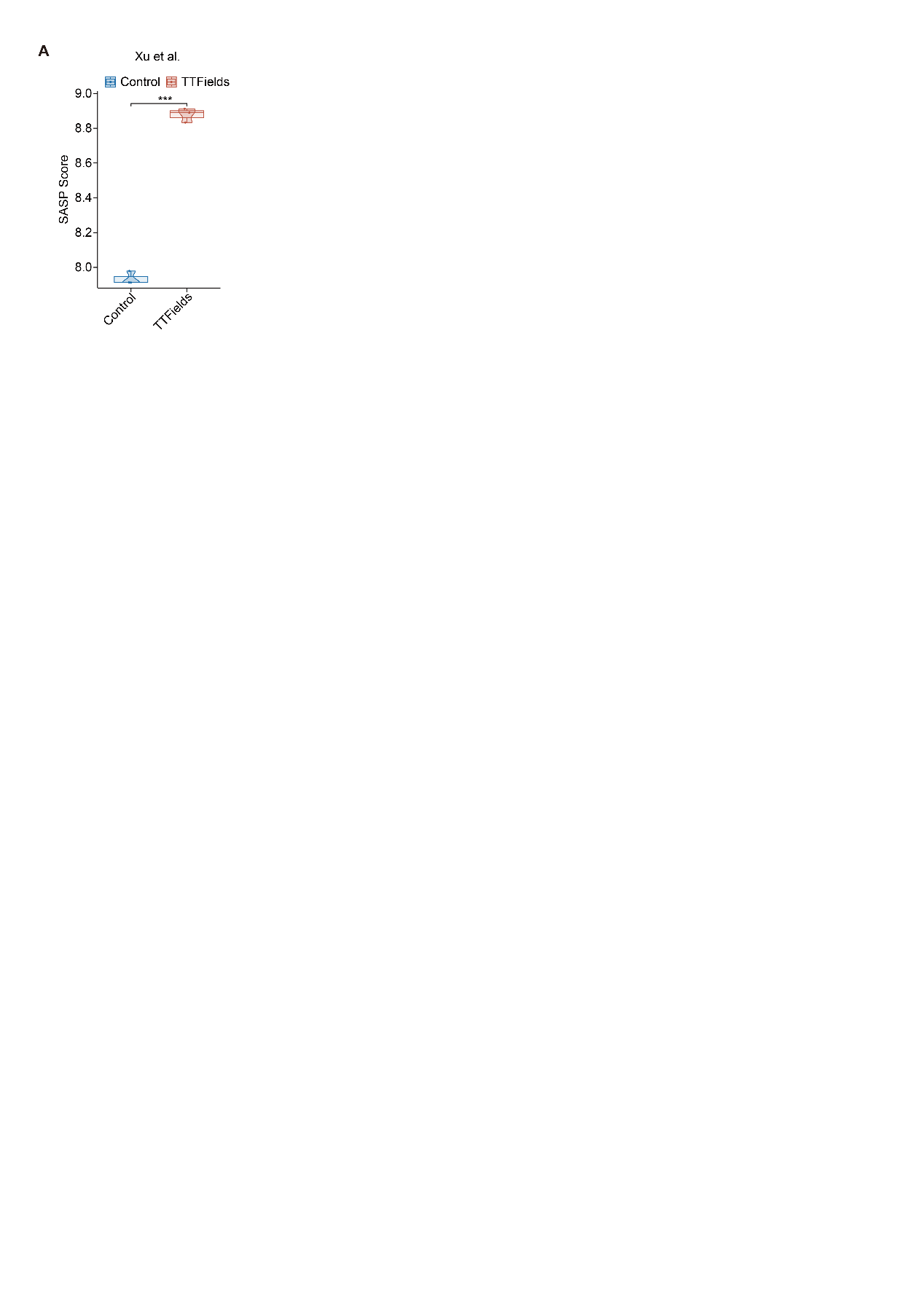


**Figure S20 related to Figure 7.** **Elevated SASP Score are enriched in GBM samples with tumor treating field (TTF) and control treatment.**

**A.** The analysis of SASP score with the indicated published dataset of GBM with TTF and control treatment (n = 3, t test). RNA-seq data for the SASP score evaluation of GBM samples receiving TTF treatment was downloaded from the supplement information of Xu’s paper (https://www.nature.com/articles/s41419-022-05127-7#Sec30).
