# Supplementary figures and images for: Profiling with senescence-associated secretory phenotype score identifies GDC-0879 as a small molecule sensitizing glioblastoma to anti-PD1
Source: Cell Death Dis. 2025 Aug 9;16(1):602. doi: 10.1038/s41419-025-07915-3 (PMC12334699; doi:10.1038/s41419-025-07915-3)

Figure S11C

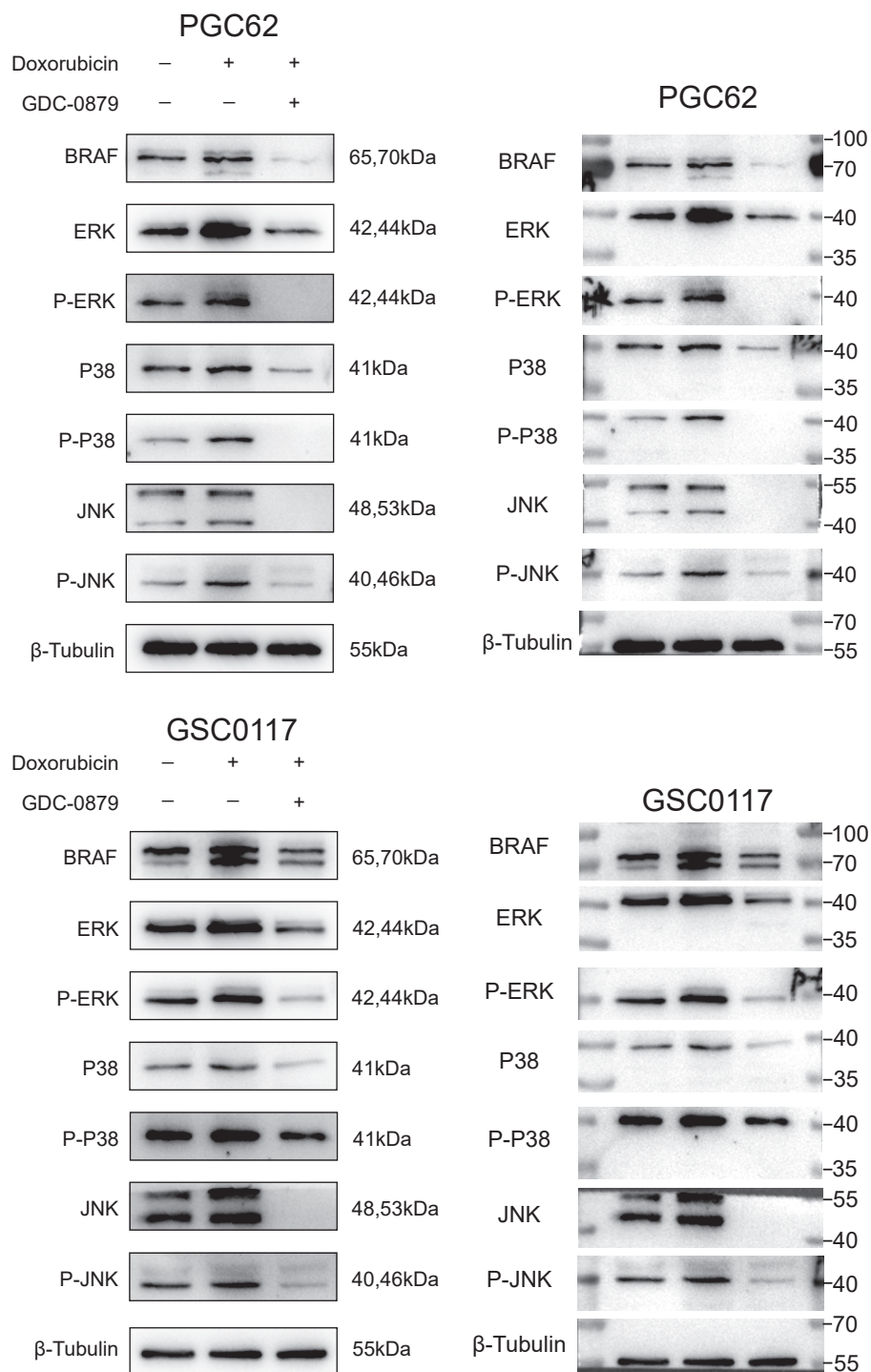

Supplement: Supplementary file 3 — Original File of Western Blot [file 41419_2025_7915_MOESM3_ESM.pdf]
